# Hospital admission on weekends for patients who have surgery and 30-day mortality in Ontario, Canada: A matched cohort study

**DOI:** 10.1371/journal.pmed.1002731

**Published:** 2019-01-29

**Authors:** James D. O’Leary, Hannah Wunsch, Anne-Marie Leo, David Levin, Asad Siddiqui, Mark W. Crawford

**Affiliations:** 1 Department of Anesthesia, University of Toronto, Toronto, Ontario, Canada; 2 Department of Anesthesia and Pain Medicine, The Hospital for Sick Children, Toronto, Ontario, Canada; 3 Institute for Clinical Evaluative Sciences, Toronto, Ontario, Canada; 4 Department of Critical Care Medicine, Sunnybrook Health Sciences, Toronto, Ontario, Canada; Massachusetts General Hospital, UNITED STATES

## Abstract

**Background:**

Healthcare interventions on weekends have been associated with increased mortality and adverse clinical outcomes, but these findings are inconsistent. We hypothesized that patients admitted to hospital on weekends who have surgery have an increased risk of death compared with patients who are admitted and have surgery on weekdays.

**Methods and findings:**

This matched cohort study included 318,202 adult patients from Ontario health administrative and demographic databases, admitted to acute care hospitals from 1 January 2005 to 31 December 2015. A total of 159,101 patients who were admitted on weekends and underwent noncardiac surgery were classified by day of surgery (weekend versus weekday) and matched 1:1 to patients who both were admitted and had surgery on a weekday (Tuesday to Thursday); matching was based on age (in years), anesthesia basic unit value for the surgical procedure, median neighborhood household income quintile, resource utilization band (a ranking system of overall morbidity), rurality of home location, year of admission, and urgency of admission. Of weekend admissions, 16.2% (25,872) were elective and 53.9% (85,744) had surgery on the weekend of admission. The primary outcome was all-cause mortality within 30 days of the date of hospital admission. The 30-day all-cause mortality for patients admitted on weekends who had noncardiac surgery was 2.6% (4,211/159,101) versus 2.5% (3,901/159,101) for those who were admitted and had surgery on weekdays (adjusted odds ratio [OR] 1.05; 95% CI 1.00 to 1.11; *P* = 0.03). However, there was significant heterogeneity in the increased odds of death according to the urgency of admission and when surgery was performed (weekend versus weekday). For urgent admissions on weekends (*n =* 133,229), there was no significant increase in odds of mortality when surgery was performed on the weekend (adjusted OR 1.02; 95% CI 0.95 to 1.09; *P* = 0.7) or on a subsequent weekday (adjusted OR 1.05; 95% CI 0.98 to 1.12; *P* = 0.2) compared to urgent admissions on weekdays. Elective admissions on weekends (*n =* 25,782) had increased risk of death both when surgery was performed on the weekend (adjusted OR 3.30; 95% CI 1.98 to 5.49; *P* < 0.001) and when surgery was performed on a subsequent weekday (adjusted OR 2.70; 95% CI 1.81 to 4.03; *P* < 0.001). The main limitations of this study were the lack of data regarding reason for admission and cause of increased time interval from admission to surgery for some cases, the small number of deaths in some subgroups (i.e., elective surgery), and the possibility of residual unmeasured confounding from increased illness severity for weekend admissions.

**Conclusions:**

When patients have surgery during their hospitalization, admission on weekends in Ontario, Canada, was associated with a small but significant proportional increase in 30-day all-cause mortality, but there was significant heterogeneity in outcomes depending on the urgency of admission and when surgery was performed. An increased risk of death was found only for elective admissions on weekends; whether this is a function of patient-level factors or represents a true weekend effect needs to be further elucidated. These findings have potential implications for resource allocation in hospitals and the redistribution of elective surgery to weekends.

## Abstract 2

In their cohort study, James Daniel O'Leary find that data stratification leads to fuller insight into the 'weekend effect' of increased mortality following hospital admission and surgery.

## Author summary

**Why was this study done?:**

- There is ongoing debate about whether a weekend effect found in most observational research studies reflects actual differences in healthcare outcomes on the weekend or is a data artifact from using administrative datasets.
- The possibility of a weekend effect continues to inform the allocation of hospital resources, healthcare policies, and clinical practices.

**What did the researchers do and find?:**

- We used provincial administrative healthcare and demographic databases in Ontario, Canada, from 2005 to 2015 to assemble a cohort of 159,101 adults who were admitted on weekends and had surgery performed during their hospital stay on the same weekend or a subsequent weekday, and who were matched directly to adults with weekday admissions and surgery.
- We found an increase in the crude mortality rate for all weekend admissions, but there was significant heterogeneity in adjusted outcomes according to the urgency of weekend admission and when surgery was performed (weekend versus weekday).
- For urgent admissions on weekends, there was no significant increase in the adjusted odds of death. However, elective admissions on weekends were associated with clinically important increases in odds of death compared with weekday admissions.

**What do these findings mean?:**

- The proportional increase in the adjusted mortality rate for weekend admissions was lower than that found in most previous observational studies using administrative data.
- However, stratification of these findings in the current study suggests that only elective admissions on weekends are associated with an increase in odds of death.
- Whether the increased risk of death found for elective admissions on weekends is a factor of increased illness severity in this population, requiring preoperative optimization, or a true weekend effect needs to be elucidated in further studies.

## Introduction

Healthcare interventions on weekends have been associated with increased mortality and adverse clinical outcomes [1–11], but these findings are inconsistent [12–15]. The overall interpretation of studies of different populations, healthcare systems, and procedures has generated much controversy and debate regarding the possibility of a “weekend effect” impacting the quality of healthcare [10,16].

Staffing of clinical specialists and technical services is typically reduced on weekends despite continued hospital admissions [17]. This change in the distribution of resources is frequently hypothesized to be a central cause of a weekend effect. However, several large-scale observational studies using healthcare data from the English National Health Service have recently provided compelling evidence that the weekend effect is unlikely to be a function of decreased specialist medical staffing [18,19]. Other explanations for the weekend effect include both patient (e.g., severity of illness, delayed presentation) and hospital (e.g., raised admission threshold, capacity, decreased diagnostic and interventional resource availability) factors. Unaccounted for differences in illness severity and indication bias almost certainly contributed to results in older observational studies that increased concern of a weekend effect [20,21]. However, organizational delays for therapeutic and diagnostic procedures are common on weekends [17], and there is substantial potential for harm when clinical care is delayed, even in otherwise healthy patients [22].

Redistribution of some surgery to the weekend has been suggested as a feasible and safe means of improving the productivity of hospitals working at capacity [23]. However, elective admissions and surgery on weekends have been associated with increased risks of adverse clinical outcomes compared with admissions and surgery on weekdays [7,24]. The aim of this study was to examine whether there is an increased risk of 30-day all-cause mortality for patients who are admitted to hospital on weekends and undergo noncardiac surgery compared with patients who are admitted and undergo surgery on weekdays, stratified by (i) when surgery was performed (weekend versus weekday) and (ii) the type of admission (elective versus urgent).

## Methods

### Ethics statement

The Research Ethics Board at The Hospital for Sick Children, Toronto (#1000055744), approved the study, and the requirement for written informed consent from study participants was waived.

### Study design

This was a matched cohort study using population-based administrative and demographic databases housed at the Institute for Clinical Evaluative Sciences (ICES) [25], specifically (i) the Discharge Abstract Database of the Canadian Institute for Health Information (CIHI) and (ii) the Ontario Registered Persons Database. These healthcare and demographic databases undergo rigorous data quality controls to ensure accuracy of data, reliability, and comparability over time [26]. This study was conducted using a prespecified analysis plan (S1 Dataset Creation and Analysis Plan) and is reported as per the RECORD guidelines (S1 Checklist).

### Study period

The study timeframe was 1 January 2005 to 31 December 2015. The start of the study period coincided with the introduction of Canadian Classification of Health Interventions (CCI) therapeutic intervention codes in the CIHI Discharge Abstract Database.

### Study population

The index event for this episode-level analysis was any hospital admission for an individual aged ≥18 years in an acute care hospital in Ontario, Canada, associated with an eligible surgical procedure in the CIHI Discharge Abstract Database performed during the same weekend or week of admission. Surgical procedures were identified using CCI therapeutic intervention codes. All eligible surgical procedures (S1 Table) identified within the study period were documented. Excluded interventions consisted of cardiothoracic or cardiology therapeutic procedures, non-surgical therapeutic interventions (e.g., dialysis), and obstetric procedures. These procedures were excluded because of population-specific differences in risk adjustment for patient-level factors, workflow, and staffing of operating rooms. Eligible admissions were classified by (i) days (weekend versus weekday) of hospital entry and of surgery and (ii) type of admission (elective versus urgent). Days were considered from midnight to midnight. The weekend was defined as Saturday or Sunday, and weekdays included Tuesday to Thursday only. Friday admissions and surgery were excluded from both groups to avoid a potential exposure misclassification with weekend clinical activity [5,27]; similarly, as time of surgery was not available for all procedures, Monday admissions were excluded from the weekday admission group to avoid risk of misclassification of admissions that occurred after midnight on Sunday night but before the start of regular working hours. Type of admission (elective versus urgent) reflects the patient’s status at the start of the admission and was identified from the admit category field in the CIHI Discharge Abstract Database; elective admissions are defined as patients who were on an elective booking list or had a scheduled admission for treatment and/or assessment. Repeat admissions within 30 days of an index event were excluded from the cohort due to risk of misclassification.

### Covariates

Demographic characteristics available included age in years, comorbidities (using Charlson Comorbidity Index with a 5-year look back), median neighborhood household income quintile (5 groups, from lowest [1] to highest [5] income), rurality of home location, and sex. Other data available included hospital local health integrated network (LHIN); length of hospital stay; anesthesia basic unit value for the surgical procedure; teaching hospital status; year of index event; mortality risk score and resource utilization band (RUB) (i.e., a ranking system of overall morbidity and health resource use: 0, nonusers; 1, healthy users; 2, users with low morbidity; 3, users with moderate morbidity; 4, users with high morbidity; and 5, users with very high morbidity), both based on the Johns Hopkins Adjusted Clinical Group case-mix system; special care unit admission (i.e., any medical or surgical intensive care or step-down unit) prior to surgery; surgical service responsible for the index surgery; and time interval from hospital admission to surgery. Relative value guides for billing have previously been used as a criterion for discriminating the physiological complexity of surgical procedures [28,29]; surgical procedures in this Ontario-based cohort were classified using anesthesia basic unit values for individual CCI therapeutic intervention codes in the 2015 Ontario Health Insurance Plan Schedule of Benefits [30]. Length of hospital stay was calculated as the difference between the admission and discharge dates in the CIHI Discharge Abstract Database.

### Outcomes

The primary outcome was all-cause mortality within 30 days of the date of hospital admission. Thirty-day all-cause mortality was determined from the Registered Persons Database.

### Statistical analysis

Both matching and regression techniques were used to mitigate confounding between weekend exposure and study outcomes. Individuals in the weekend and weekday admission groups were classified by day (weekend versus weekday) of surgery and then matched directly using an exact matching technique (ratio 1:1) on 7 variables (age in years, anesthesia basic unit value for the surgical procedure, median neighborhood household income, RUB, rural home location, year of admission, and urgency of admission). Descriptive statistics and unadjusted differences were determined as appropriate for the data distribution in the study groups. Generalized estimating equation–based multivariable logistic regression models for matched pairs nested within hospital clusters were used to estimate the adjusted association between weekend exposure (independent variable) and 30-day all-cause mortality (dependent variable). Covariates tested (Charlson Comorbidity Index, hospital LHIN, sex, teaching hospital status, mortality risk score, preoperative special care unit admission, and responsible surgical service) in regression models were chosen based on their standardized differences between weekend and weekday admission groups. Subgroup analyses were performed based on day of surgery (weekend versus weekday) and type of admission (elective versus urgent). The comparison groups for all analyses were matched patients who were admitted and had surgery on a weekday. Results were summarized using odds ratio (OR) estimates and 95% confidence intervals (CIs). In a sensitivity analysis, adjusted ORs of 30-day all-cause mortality from date of surgery (instead of date of hospital admission) were calculated. Statistical significance was defined as 2-tailed *P* < 0.05.

In response to peer review comments, several adjustments were made to both the covariates included in the cohort and the analyses performed. First, the following additional covariates were included in the analyses: the Charlson Comorbidity Index, a mortality risk score (based on the Johns Hopkins Adjusted Clinical Group case-mix system), preoperative admission to a special care unit, and responsible surgical service. These variables were included in models to provide more accurate clinical information on comorbidities, illness severity, and perioperative risk. Second, models tested matched pairs nested within hospital clusters to account for assortative mixing of patients in hospitals. Third, additional sensitivity analyses were performed to test whether the increased time interval to surgery observed on weekends was contributing to differences between groups, specifically we conducted all analyses (overall and subgroup) also adjusting for the time interval from admission to surgery and, where appropriate, including an interaction term between time to surgery and admission type (elective versus urgent).

All statistical analyses were performed with SAS 9.4 (SAS Institute, Cary, NC).

## Results

### Patient characteristics

A total of 1,366,221 eligible hospital admissions for patients who underwent noncardiac surgery in Ontario, Canada, during the 11-year study period were identified (S2 Table). From 212,387 admissions on weekends, 159,101 (74.9%) were classified by day of surgery and matched directly (1:1) to patients with a weekday admission and surgery. Characteristics of matched and unmatched weekend admissions are summarized in S3 Table. In the group of 159,101 weekend admissions, 85,744 (53.9%) had a surgical procedure performed on the same weekend and 73,357 (46.1%) had a surgical procedure performed on a subsequent weekday. A total of 25,872 (16.2%) weekend admissions were elective. Characteristics of the weekend and weekday admission groups in the matched cohort are summarized in Table 1 and are categorized by the day (weekend versus weekday) of surgery in S4 and S5 Tables. Of note, patients admitted on weekends were more likely to be male, to be admitted to a non-teaching hospital, and to have a longer interval between hospital admission and surgery than patients admitted on weekdays (Table 1). Among weekend admissions, patients who had their surgery performed on the same weekend were more likely to be younger, be female, be admitted to hospital urgently, live in a lower income neighborhood, have a lower RUB score, and have a higher Charlson Comorbidity Index (S4 and S5 Tables) than those whose surgery was performed on a subsequent weekday. The type and percentage of the 10 most common surgical procedures in each of the matched groups, which account for over 60% of surgical procedures performed in the matched cohort, are summarized in S6 Table.

**Table 1:** Characteristics of all eligible admissions on weekends for patients who had noncardiac surgery in Ontario hospitals between January 2005 and December 2015 matched directly to weekday admissions.

Characteristic	Weekend admission *N* = 159,101	Weekday admission *N* = 159,101	*P* value^a^
Age category, *n* (%)			NA
18 to 49 years	57,869 (36.4)	57,822 (36.3)	
50 to 64 years	38,242 (24.0)	38,273 (24.1)	
≥65 years	62,990 (39.6)	63,006 (39.6)	
Male, *n* (%)	81,273 (51.1)	72,987 (45.9)	<0.001
Median neighborhood household income quintile, *n* (%)			NA
Missing	99 (0.1)	99 (0.1)	
1—lowest	33,016 (20.8)	33,016 (20.8)	
2	31,881 (20.0)	31,881 (20.0)	
3	31,157 (19.6)	31,157 (19.6)	
4	32,146 (20.2)	32,146 (20.2)	
5	30,802 (19.4)	30,802 (19.4)	
Rural home location, *n* (%)	11,922 (7.5)	11,922 (7.5)	NA
Resource utilization band^b^, *n* (%)			NA
0—lowest	≤5 (S)	≤5 (S)	
1	8–12 (S)	8–12 (S)	
2	9,250 (5.8)	9,250 (5.8)	
3	56,420 (35.5)	56,420 (35.5)	
4	41,564 (26.1)	41,564 (26.1)	
5	51,855 (32.6)	51,855 (32.6)	
Charlson Comorbidity Index, *n* (%)			0.02
0	121,124 (76.1)	121,754 (76.5)	
1	12,366 (7.8)	12,258 (7.7)	
≥2	25,611 (16.1)	25,089 (15.8)	
Mortality risk score^c^, mean ± SD	72.43 ± 27.45	72.45 ± 27.14	0.8
Year of admission, *n* (%)			NA
2005	14,195 (8.9)	14,195 (8.9)	
2006	13,905 (8.7)	13,905 (8.7)	
2007	13,975 (8.8)	13,975 (8.8)	
2008	14,108 (8.9)	14,108 (8.9)	
2009	14,213 (8.9)	14,213 (8.9)	
2010	14,017 (8.8)	14,017 (8.8)	
2011	14,416 (9.1)	14,416 (9.1)	
2012	14,764 (9.3)	14,764 (9.3)	
2013	14,968 (9.4)	14,968 (9.4)	
2014	15,422 (9.7)	15,422 (9.7)	
2015	15,118 (9.5)	15,118 (9.5)	
Elective admission, *n* (%)	25,872 (16.3)	25,872 (16.3)	NA
Admission to a teaching hospital, *n* (%)	47,780 (30.0)	49,858 (31.3)	<0.001
Surgical procedures with ≥8 OHIP anesthesia basic units, *n* (%)	14,007 (8.8)	14,007 (8.8)	NA
Admitted to a special care unit prior to surgery, *n* (%)	15,228 (9.6%)	14,874 (9.3%)	0.03
Days from admission to surgery, mean ± SD	1.0 ± 1.2	0.4 ± 0.6	<0.001
Length of hospital stay, mean ± SD	6.5 ± 10.8	5.7 ± 11.6	<0.001

Variables used for exact matching were age in years, anesthesia basic unit value for the surgical procedure, median neighborhood household income, resource utilization band, rural home location, year of admission, and urgency of admission.

^a^*P* values not reported for variables used in exact matching of study groups.

^b^Resource utilization band is a ranking system of overall morbidity and health resource use based on the Johns Hopkins Adjusted Clinical Group case-mix system.

^c^Mortality risk score based on the Johns Hopkins Adjusted Clinical Group case-mix system.

NA, not applicable; OHIP, Ontario Health Insurance Plan; S, suppressed percentage (cell counts < 6 cannot be reported); SD, standard deviation.

### Weekend admission for patients who had noncardiac surgery and 30-day all-cause mortality

The 30-day all-cause mortality for patients who had an admission on weekends and subsequent noncardiac surgery was 2.6% versus 2.5% for those who were admitted on weekdays. After adjusting for confounding factors (Charlson Comorbidity Index, hospital LHIN, sex, teaching hospital status, mortality risk score, preoperative special care unit admission, and responsible surgical service), there was an increased odds of 30-day all-cause mortality for patients admitted on weekends compared with those who were admitted and had surgery on weekdays (adjusted OR 1.05; 95% CI 1.00 to 1.11; *P* = 0.03) (Table 2).

**Table 2:** Thirty-day all-cause mortality for patients admitted on weekends who had noncardiac surgery compared with reference admissions (i.e., matched patients who were admitted and had surgery on weekdays), classified by day of surgery (weekend and weekday) and urgency.

Admission type	30-day all-cause mortality, *n/N* (%)		Adjusted odds ratio (95% CI)
	Weekend admission	Weekday admission	
All weekend admissions	4,211/159,101 (2.6)	3,901/159,101 (2.5)	1.05 (1.00 to 1.11)
Weekend admission and weekend surgery			
Urgent	1,922/79,339 (2.4)	1,848/79,339 (2.3)	1.02 (0.95 to 1.09)
Elective	62/6,405 (1.0)	22/6,405 (0.3)	3.30 (1.98 to 5.49)^a^
Subtotal (urgent + elective)	1,984/85,744 (2.3)	1,870/85,744 (2.2)	1.04 (0.97 to 1.11)
Weekend admission and weekday surgery			
Urgent	2,110/53,890 (3.9)	1,995/53,890 (3.7)	1.05 (0.98 to 1.12)
Elective	117/19,467 (0.6)	36/19,467 (0.2)	2.70 (1.81 to 4.03)^b^
Subtotal (urgent + elective)	2,227/73,357 (3.0)	2,031/73,357 (2.8)	1.06 (1.00 to 1.14)

Variables used for exact matching were age in years, anesthesia basic unit value for the surgical procedure, median neighborhood household income, resource utilization band, rural home location, year of admission, and urgency of admission. Covariates adjusted for in models were Charlson Comorbidity Index, local health integrated network, sex, teaching hospital status, mortality risk score, preoperative special care unit admission, and responsible surgical service.

^a^Not adjusted for Local Health Integrated Network and responsible surgical service due to lack of model convergence.

^b^Not adjusted for responsible surgical service due to lack of model convergence.

CI, confidence interval.

### Weekend and weekday surgery for weekend admissions and 30-day all-cause mortality

When weekend admissions were classified by the day of surgery (weekend versus weekday), 30-day all-cause mortality for patients who had surgery on the weekend was 2.3% versus 3.0% when surgery was performed on a subsequent weekday (Table 2).

There were no statistical differences in crude mortality rates (2.3% versus 2.2%; *P* = 0.07) or in the odds of 30-day all-cause mortality (adjusted OR 1.04; 95% CI 0.97 to 1.11; *P* = 0.3) for patients who were admitted and had surgery performed on the weekend compared with patients who were admitted and had surgery performed on a weekday (Table 2). Patients who were admitted on the weekend and had surgery performed on a subsequent weekday had an increased crude mortality compared with those who were admitted and had surgery on a weekday (3.0% versus 2.8%; *P* = 0.003), but, in adjusted models, there was no increase in the odds of 30-day all-cause mortality (adjusted OR 1.06; 95% CI 1.00 to 1.14; *P* = 0.07) (Table 2).

### Urgency of weekend admission and 30-day all-cause mortality

In the matched cohort, 16.2% (25,872/159,101) of admissions on weekends were elective, and 75.2% (19,467/25,872) of these had surgery performed on a subsequent weekday. The characteristics of elective and urgent admissions in the matched cohort are summarized in S7 Table and S8 Table, respectively.

Among urgent admissions, there was no increase in the adjusted odds of 30-day all-cause mortality when surgery was performed on the weekend (adjusted OR 1.02; 95% CI 0.95 to 1.09; *P* < 0.7) or when surgery was performed on a subsequent weekday (adjusted OR 1.05; 95% CI 0.98 to 1.12; *P* = 0.2), each compared with weekday admissions.

Elective admissions on weekends had the lowest crude mortality rates in the cohort, but, when compared with elective weekday admissions, they had the highest adjusted ORs of 30-day all-cause mortality among admission types (Table 2). For elective weekend admissions, there was an increased odds of 30-day all-cause mortality compared with elective weekday admissions when surgery was performed on the same weekend (adjusted OR 3.30; 95% CI 1.98 to 5.49; *P* < 0.001) or on a subsequent weekday (adjusted OR 2.70; 95% CI 1.81 to 4.03; *P* < 0.001).

### Sensitivity analysis: Increased time interval from admission to surgery on weekends and 30-day all-cause mortality

We measured 30-day mortality from date of surgery (instead of from date of hospital admission) to test for a survivor bias; there was a small increase in the odds of mortality for weekend admissions overall and for weekend admissions where surgery was not performed on the same weekend (S9 Table). In a post hoc sensitivity analysis, all models were additionally adjusted for the interval (in days) from admission to surgery (S10 Table). There was no meaningful change in the magnitude or direction of odds of death for weekend admissions. Urgent weekend admissions for which surgery was performed on a subsequent weekday had no increase in odds of death (adjusted OR 0.97; 95% CI 0.89 to 1.06; *P* = 0.5) compared with urgent weekday admissions; elective weekend admissions for which surgery was performed on a subsequent weekday maintained a large increase in odds of 30-day all-cause mortality (adjusted OR 2.39; 95% CI 1.43 to 4.00; *P* < 0.001) compared with elective weekday admissions.

## Discussion

This study of 159,101 matched hospital admissions of patients who subsequently had surgery in Ontario, Canada, showed a small but significant proportional increase in 30-day all-cause mortality for patients who were admitted on weekends compared with patients admitted on weekdays, but with significant heterogeneity in outcomes according to the urgency of admission and when surgery was performed (weekend versus weekday). Elective admissions on weekends (16% of the cohort) were associated with the highest relative increases in crude and adjusted odds of death, regardless of whether surgery was performed on the weekend or on a subsequent weekday. For urgent weekend admissions, the study found no increase in odds of death when surgery was performed either on the weekend of admission or on a subsequent weekday.

Similarly to previous large-scale studies using administrative databases, the current study included both elective and urgent hospital admissions in the cohort [9], and matched or adjusted for multiple socioeconomic, demographic, and clinical covariates in the analysis [2]. The estimate of an overall increased odds of death of 5% for all weekend admissions is lower than that found in previous studies using data commonly available to administrative datasets [2,15]. However, as demonstrated by Walker et al. [15], it has become increasingly evident that mortality estimates using administrative data cannot comprehensively account for differences in acuity of illness or disease severity that exist between patients who are admitted to hospitals on weekends and weekdays, and these mortality estimates are likely overestimates [20]. Walker et al. demonstrated an 8%–9% increase in risk of death for emergent weekend admissions compared with midweek admissions using administrative data only, but found that adjusting for abnormalities in commonly used laboratory tests explained 33% to 52% of excess mortality on weekends. There are several possible reasons for the overall increase in odds of death for weekend admissions in this cohort, but our findings suggest that the urgency of admission is an important effect modifier of postoperative mortality among weekend admissions.

Few studies have examined whether there is evidence of a weekend effect for elective admissions. In general, studies of a weekend effect for elective procedures or admissions find persistent evidence of an increased mortality risk. In a large multicenter English study, Aylin et al. found that elective surgical procedures performed on weekends had an 82% higher odds of death compared with elective surgical procedures performed on Mondays [5]. In another English study, including 127,562 elective surgical admissions on weekends, Mohammed et al. found an increased odds of death for elective weekend admissions compared with emergent weekend admissions, 32% and 9%, respectively [24]. Similarly to this study, using data from Ontario, McIsaac et al. demonstrated a significant increase in mortality on weekends for major elective noncardiac surgery, with a 51% higher odds of postoperative death [7].

The findings of the current study are novel. They indicate that the OR of mortality is increased for elective admissions on weekends—independent of whether surgery is performed on the weekend or on a subsequent weekday—but not for urgent admissions on weekends. This finding was found to be robust in sensitivity analyses but was influenced by the increased time from admission to surgery that occurs on weekends. There are many reasons why the time from admission to surgery can be increased on weekends, and these are often classified as organizational and patient factors. Organizational delays for therapeutic and diagnostic procedures are among the most common reasons for inappropriate hospital stay [31], but considering the raised threshold for hospital admission on weekends [21], it is likely that patients admitted on weekends in this cohort also had increased severity of illness and additional comorbidities. Preoperative optimization of these patient factors could account for the relative decrease in the OR of mortality observed in the cohort of elective weekend admissions where patients had surgery performed on a subsequent weekday, and for the sensitivity of this finding to the effects of increased time from admission to surgery. Nonetheless, delayed surgery for any reason can still be a significant risk factor for perioperative mortality. For example, a delay of 1 day between admission and surgery for patients with fractured neck of femur increases the odds of in-hospital death [32], and even for otherwise healthy patients, delayed surgery—albeit a delay of longer duration—can be associated with increased risk of death [22].

This study has several strengths, including the use of a large provincial population and healthcare administrative databases that include all admissions and surgical procedures performed in the province. As a consequence, bias resulting from missing data is unlikely in this study. In addition, the large sampling frame allowed imbalances between groups to be reduced by matching directly and simultaneously on multiple covariates, avoiding the limitations of using propensity scoring [33]. The accuracy and comprehensiveness of administrative databases for some clinical information may be diminished relative to clinical databases [34]; nonetheless, the reporting of mortality can be similar between administrative and clinical databases [35].

This study has some limitations. First, we are unable to elucidate the causes of increased time between admission and surgery experienced by patients admitted on weekends (i.e., whether these were delays indicated by clinical reasons, an evolving clinical problem not initially necessitating surgery on admission, or preoperative optimization, or whether surgery was indicated from the start of the admission and delays were due to staffing and resource availability). Second, consistent with previous studies [1], days in this study were defined from midnight to midnight. This does introduce a risk of misclassification for procedures performed after midnight on Sunday night, which were classified as weekday surgeries yet were performed with weekend staff. Thus, it is possible that some increase in mortality would be attributable to surgery that is performed on the weekend if these cases were reclassified. However, such cases likely represented a very small proportion of the total surgical cases we assessed. Illness burden should also be considered as a potential effect modifier when analyzing the weekend effect [7]. Third, although we accounted for complexity of surgical procedures, comorbidities, and multiple other patient and demographic covariates that can influence clinical outcomes, there is still potential for unmeasured confounding, including from illness severity and clustering of patients who are admitted on weekends [15,21].

### Conclusions

When patients had surgery during their hospitalization, admission on weekends in Ontario, Canada, was associated with a small but significant proportional increase in 30-day all-cause mortality, but there was significant heterogeneity in outcomes depending on the urgency of admission and when surgery was performed. The increased odds of mortality was found only among elective admissions on weekends; whether this is a factor of increased illness severity requiring preoperative optimization or represents a true weekend effect needs to be further elucidated. These findings have potential implications for resource allocation in hospitals and the redistribution of elective surgery to weekends.

## Supporting information

S1 Checklist RECORD checklist. (DOCX)

S1 Dataset Creation and Analysis Plan Dataset creation and data analysis plan, edited for clarity. (DOCX)

S1 Table Surgical procedure definition. (DOCX)

S2 Table Characteristics of all adult admissions with noncardiac surgery performed in Ontario hospitals between January 2005 and December 2015, classified by day (weekend or weekday) of admission and surgery. (DOCX)

S3 Table Characteristics of matched and unmatched weekend admissions with noncardiac surgery performed in Ontario hospitals between January 2005 and December 2015. (DOCX)

S4 Table Characteristics of eligible weekend admissions with weekend noncardiac surgery performed in Ontario hospitals between January 2005 and December 2015 matched directly to weekday admissions on age, complexity of surgical procedure, median neighborhood household income, resource utilization band, year of admission, and urgency of admission. (DOCX)

S5 Table Characteristics of eligible weekend admissions with weekday noncardiac surgery performed in Ontario hospitals between January 2005 and December 2015 matched directly to weekday admissions on age, complexity of surgical procedure, median neighborhood household income, resource utilization band, year of admission, and urgency of admission. (DOCX)

S6 Table Frequency of the 10 most common noncardiac surgical procedures performed for weekend and weekday admissions in the matched cohort, classified by day of surgery for weekend admissions (weekday or weekend), type of admission (elective or urgent), and sex. (DOCX)

S7 Table Characteristics of elective admissions included in the matched cohorts, classified by day of surgery (weekend or weekday). (DOCX)

S8 Table Characteristics of urgent admissions included in the matched cohorts, classified by day of surgery (weekend or weekday). (DOCX)

S9 Table Adjusted odds ratios of 30-day all-cause mortality for patients admitted on weekends who had noncardiac surgery compared with reference admissions, measured from day of admission and from day of surgery. (DOCX)

S10 Table Adjusted odds ratios of 30-day all-cause mortality for patients admitted on weekends who had noncardiac surgery compared with reference admissions, with and without adjusting for the time interval from admission to surgery and with an interaction term between time to surgery and urgency of admission. (DOCX)

## Abbreviations

CCI: Canadian Classification of Health Interventions
CIHI: Canadian Institute for Health Information
ICES: Institute for Clinical Evaluative Sciences
LHIN: local health integrated network
OR: odds ratio
RUB: resource utilization band
