# Supplementary material for: Hospital admission on weekends for patients who have surgery and 30-day mortality in Ontario, Canada: A matched cohort study
Source: PLoS Med. 2019 Jan 29;16(1):e1002731. doi: 10.1371/journal.pmed.1002731 (PMC6350956; doi:10.1371/journal.pmed.1002731)
Supplement: S1 Checklist — (DOCX) [file pmed.1002731.s001.docx]

**S1 RECORD Checklist.** RECORD Checklist

|  | **Item No.** | **STROBE items** | **RECORD items** | **Location in manuscript where items are reported** |
| --- | --- | --- | --- | --- |
| **Title and abstract** | | | | |
|  | 1 | (a) Indicate the study’s design with a commonly used term in the title or the abstract (b) Provide in the abstract an informative and balanced summary of what was done and what was found | RECORD 1.1: The type of data used should be specified in the title or abstract. When possible, the name of the databases used should be included.  RECORD 1.2: If applicable, the geographic region and timeframe within which the study took place should be reported in the title or abstract.  RECORD 1.3: If linkage between databases was conducted for the study, this should be clearly stated in the title or abstract. | Title and/or abstract contain:   - 1. The type of data used (“health administrative and demographic databases”)   2. 1.2 The geographic region (Ontario) and timeframe (“from 1 January 2005 to 31 December 2015”)   3. No linkage was conducted for this study |
| **Introduction** | | | | |
| Background rationale | 2 | Explain the scientific background and rationale for the investigation being reported | | Introduction, Paragraph 2 and 3, including:  “Staffing of clinical specialists and technical services are typically reduced on weekends despite continued hospital admissions. This change in the distribution of resources is frequently hypothesized to be a central cause for a weekend effect.”  “Unaccounted for illness severity and indication bias on weekends have almost certainly contributed to older observational studies that increased concern of a weekend effect. However, organizational delays for therapeutic and diagnostic procedures are common on weekends, and there is substantial potential for harm when clinical care is delayed, even in otherwise healthy patients.” |
| Objectives | 3 | State specific objectives, including any prespecified hypotheses | | Introduction: “The aims of this study were to examine whether there is an increased risk of 30-day all-cause mortality for patients who are admitted to hospital on weekends and undergo noncardiac surgery compared with patients who are admitted and undergo surgery on weekdays, stratified by i) when (weekend vs. weekday) surgery was performed and ii) the type (elective vs. urgent) of admission.” |
| **Methods** | | | | |
| Study Design | 4 | Present key elements of study design early in the paper | | Methods, Paragraphs 1 & 3 – provincial-based matched cohort study. |
| Setting | 5 | Describe the setting, locations, and relevant dates, including periods of recruitment, exposure, follow-up, and data collection | | Methods:  Paragraph 2 (Design – setting, locations)  Paragraph 3 (Period – relevant dates)  Paragraph 4 (Population – exposure detailed) |
| Participants | 6 | *(a) Cohort study* - Give the eligibility criteria, and the sources and methods of selection of participants. Describe methods of follow-up  *Case-control study* - Give the eligibility criteria, and the sources and methods of case ascertainment and control selection. Give the rationale for the choice of cases and controls  *Cross-sectional study* - Give the eligibility criteria, and the sources and methods of selection of participants  *(b) Cohort study* - For matched studies, give matching criteria and number of exposed and unexposed  *Case-control study* - For matched studies, give matching criteria and the number of controls per case | RECORD 6.1: The methods of study population selection (such as codes or algorithms used to identify subjects) should be listed in detail. If this is not possible, an explanation should be provided.  RECORD 6.2: Any validation studies of the codes or algorithms used to select the population should be referenced. If validation was conducted for this study and not published elsewhere, detailed methods and results should be provided.  RECORD 6.3: If the study involved linkage of databases, consider use of a flow diagram or other graphical display to demonstrate the data linkage process, including the number of individuals with linked data at each stage. | 1. Eligibility criteria are in Methods, Paragraph (Study Population), sources are described in Methods, Paragraph 2. 2. Matching criteria are described in Methods (Paragraph 7 – Statistical Analysis)   6.1 Not applicable  6.2 Not applicable  6.3 Not applicable |
| Variables | 7 | Clearly define all outcomes, exposures, predictors, potential confounders, and effect modifiers. Give diagnostic criteria, if applicable. | RECORD 7.1: A complete list of codes and algorithms used to classify exposures, outcomes, confounders, and effect modifiers should be provided. If these cannot be reported, an explanation should be provided. | Exposure: Methods, Paragraph 4 defines and classifies the exposure groups.  Confounders: Methods, Paragraph 5 includes all covariates tested.  Outcomes: Methods, Paragraph 6 defines the outcome measure “all-cause mortality within 30 days of the date of hospital admission”  7.1 Eligible surgical procedures and Canadian Classification of Health Interventions (CCI) therapeutic intervention codes are listed in S1 Table. |
| Data sources/ measurement | 8^*^ | For each variable of interest, give sources of data and details of methods of assessment (measurement).  Describe comparability of assessment methods if there is more than one group | | The source of the primary outcome measure is included in Methods, Paragraph 6 “Thirty-day all-cause mortality was determined from the Registered Persons Database.” |
| Bias | 9 | Describe any efforts to address potential sources of bias | | Methods to match and adjust on covariates, are described in Methods, Paragraph 7. |
| Study size | 10 | Explain how the study size was arrived at | | Methods, Paragraph 4 – The study is population-based, all eligible individuals were included. “The index event was any hospital admission for individuals aged ≥18 years in acute-care hospitals in Ontario, Canada, associated with an eligible surgical procedure in the CIHI discharge abstract database performed during the same weekend or week of admission.” |
| Quantitative variables | 11 | Explain how quantitative variables were handled in the analyses. If applicable, describe which groupings were chosen, and why | | How variables were classified or defined are described in Methods, Paragraph 5. How they were handled in the analysis is described in Methods, Paragraph 7, “Generalized estimating equation (GEE)-based multivariable logistic regression models for matched pairs nested within hospital clusters were used to estimate the adjusted association between weekend exposure (independent variable) with primary and 30-day all-cause mortality (dependent variable). Covariates tested (Charlson Comorbidity Index, hospital LHIN, sex, teaching hospital status, mortality risk score, preoperative special care unit admission, and responsible surgical service) in regression models were chosen based on their standardized differences between weekend and weekday groups. Subgroup analyses were performed based on day of surgery (weekend vs. weekday) and type of admission (elective vs. urgent). The comparison groups for all analyses used matched patients who were admitted and had surgery on a weekday.” |
| Statistical methods | 12 | (a) Describe all statistical methods, including those used to control for confounding  (b) Describe any methods used to examine subgroups and interactions  (c) Explain how missing data were addressed  (d) *Cohort study* - If applicable, explain how loss to follow-up was addressed  *Case-control study* - If applicable, explain how matching of cases and controls was addressed  *Cross-sectional study* - If applicable, describe analytical methods taking account of sampling strategy  (e) Describe any sensitivity analyses | | a) Methods, Paragraphs 7 details all statistical analyses, including those used to control for confounding factors.  b) Methods, Paragraph 7  – subgroups. “Subgroup analyses were performed based on day of surgery (weekend vs. weekday) and type of admission (elective vs. urgent).”  – survivor bias. “In a sensitivity analysis of 30-day all-cause mortality from date of surgery (instead of date of hospital admission), adjusted odds ratios for both outcome measures were calculated.”  – survivor bias. “Third, additional sensitivity analyses were performed to test whether the increased time interval to surgery observed on weekends was contributing to differences between groups, specifically we conducted all analyses (overall and subgroup) also adjusting for the time interval from admission to surgery and, where appropriate, including an interaction term between time to surgery and admission type (elective vs urgent).”  c) The data quality is addressed in Methods, Paragraph 2 “These healthcare and demographic databases undergo rigorous data quality controls to ensure accuracy of data, reliability, and comparability over time.” And Discussion, Paragraph 5 “This study has several strengths, including the use of a large provincial population and healthcare administrative databases that include all surgical admissions and procedures performed in the province. As a consequence, bias resulting from missing data is unlikely in this study.”  d) Not applicable  e) Methods:  Paragraph 7 “Third, additional sensitivity analyses were performed to test whether the increased time interval to surgery observed on weekends was contributing to differences between groups, specifically we conducted all analyses (overall and subgroup) also adjusting for the time interval from admission to surgery and, where appropriate, including an interaction term between time to surgery and admission type (elective vs urgent).” |
| Data access and cleaning methods |  | RECORD 12.1: Authors should describe the extent to which the investigators had access to the database population used to create the study population.  RECORD 12.2: Authors should provide information on the data cleaning methods used in the study. | | 12.1 The use of data from Institute of Clinical Evaluative Sciences is detailed in the footnote “This study made use of de-identified data from the ICES Data Repository, which is managed by the Institute for Clinical Evaluative Sciences with support from its funders and partners: Canada’s Strategy for Patient-Oriented Research (SPOR), the Ontario SPOR Support Unit, the Canadian Institutes of Health Research and the Government of Ontario.” Data are made available on a study-specific basis only.  12.2 Data cleaning methods were not used at a study-level, data quality of databases held at the Institute of Clinical Evaluative Sciences is addressed in the Methods, Paragraph 2. |
| Linkage |  | RECORD 12.3: State whether the study included person-level, institutional-level, or other data linkage across two or more databases. The methods of linkage and methods of linkage quality evaluation should be provided. | | Not applicable. No linkage was performed for the purposes of this study. |
| **Results** | | | | |
| Participants | 13 | (a) Report the numbers of individuals at each stage of the study (*e.g.*, numbers potentially eligible, examined for eligibility, confirmed eligible, included in the study, completing follow-up, and analysed)  (b) Give reasons for non-participation at each stage.  (c) Consider use of a flow diagram | RECORD 13.1: Describe in detail the selection of the persons included in the study (*i.e.,* study population selection) including filtering based on data quality, data availability and linkage. The selection of included persons can be described in the text and/or by means of the study flow diagram. | 1. Results, Paragraph 1 “A total of 1,366,221 eligible hospital admissions for patients who underwent noncardiac surgery in Ontario, Canada, during the 11-year study period were identified (S2 Table). From 212,387 admissions on weekends, 159,101 (74.9%) were classified by day of surgery and matched directly (1:1) to patients with a weekday admission and surgery. Characteristics of matched and unmatched weekend admissions are summarized in S3 Table. In the weekend group of 159,101 admissions, 85,744 (53.9%) had a surgical procedure performed on the same weekend and 73,357 (46.1%) had a surgical procedure performed on a subsequent weekday. A total of 25,872 (16.2%) of weekend admissions were elective.” 2. Not applicable 3. Not included, the matched study cohort creation is described in Results, Paragraph 1. |
| Descriptive data | 14 | (a) Give characteristics of study participants (*e.g.*, demographic, clinical, social) and information on exposures and potential confounders  (b) Indicate the number of participants with missing data for each variable of interest  (c) *Cohort study* - summarise follow-up time (*e.g.*, average and total amount) | | a) Results, Paragraph 1/Table 1.  b) Not applicable.  c) Not applicable – follow-up was confined to the definition of the outcome measure “30 days of the date of hospital admission”. |
| Outcome data | 15 | *Cohort study* - Report numbers of outcome events or summary measures over time  *Case-control study* - Report numbers in each exposure category, or summary measures of exposure  *Cross-sectional study* - Report numbers of outcome events or summary measures | | Results, Paragraphs 2, 3, and Table 2.  Paragraph 2: “The 30-day all-cause mortality for patients who had an admission on weekends and subsequent noncardiac surgery was 2.6% vs. 2.5% for those who were admitted on weekdays.”  Paragraph 3: “When weekend admissions were classified by the day of surgery (weekend vs. weekday), 30-day all-cause mortality for patients who had surgery on weekends was 2.3% vs. 3.0% when surgery was performed on weekdays (Table 2).” |
| Main results | 16 | (a) Give unadjusted estimates and, if applicable, confounder-adjusted estimates and their precision (e.g., 95% confidence interval). Make clear which confounders were adjusted for and why they were included  (b) Report category boundaries when continuous variables were categorized  (c) If relevant, consider translating estimates of relative risk into absolute risk for a meaningful time period | | Table 2, S9 Table, S10 Table |
| Other analyses | 17 | Report other analyses done—e.g., analyses of subgroups and interactions, and sensitivity analyses | | Results: Details of the analyses of subgroups (Weekend and weekday surgery for weekend admissions) and sensitivity analyses (Exclusion of elective surgical patients admitted on the weekend; adjustment for time interval to surgery) are provided in the main text and supplementary tables. |
| **Discussion** | | | | |
| Key results | 18 | Summarise key results with reference to study objectives | | Discussion, Paragraph 1 |
| Limitations | 19 | Discuss limitations of the study, taking into account sources of potential bias or imprecision. Discuss both direction and magnitude of any potential bias | RECORD 19.1: Discuss the implications of using data that were not created or collected to answer the specific research question(s). Include discussion of misclassification bias, unmeasured confounding, missing data, and changing eligibility over time, as they pertain to the study being reported. | Discussion, Paragraph 2, 3, 5 – Strengths and limitations are discussed.  Limitations are discussed in detail:  Discussion, Paragraph 2: “However, as demonstrated by Walker *et al*., it has become increasingly evident that mortality estimates using administrative data may not comprehensively account for differences in acuity of illness or disease severity that exist between patients who are admitted to hospitals on weekends and weekdays, and these are likely overestimates.”    Discussion, Paragraph 6 “First, we are unable to elucidate the causes of increased time between admission and surgery experienced by patients admitted on weekends (i.e., whether these were delays indicated by clinical reasons, an evolving clinical problem not initially necessitating surgery on admission, preoperative optimization, or whether surgery was indicated from the start of the admission and delays were due to staffing and resource availability).”  Discussion, Paragraph 6 “Second, consistent with previous studies [1], days in this study were defined from midnight to midnight. This does introduce a risk of misclassification for procedures performed after midnight on Sunday night, which were classified as weekday surgeries yet were performed with weekend staff. Thus, it is possible that some increase in mortality may be attributable to surgery that is performed on the weekend if these cases were to be reclassified. However, this likely represented a very small proportion of the total surgical cases we assessed. Illness burden should also be considered as a potential effect modifier when analyzing the weekend effect.”  Discussion, Paragraph 6: “Third, although we accounted for complexity of surgical procedures, comorbidities, and multiple other patient and demographic covariates that can influence clinical outcomes, there is still potential for unmeasured confounding, including from illness severity and clustering of patients who are admitted on weekends.”  19.1 The implications of using data that were not created or collected to answer the specific research question are addressed in Discussion, Paragraph 5, including the sensitivity analysis of survivor bias. “Accuracy and comprehensiveness of administrative databases for some clinical information may be diminished relative to clinical databases [34]; nonetheless, the reporting of mortality can be similar between administrative and clinical databases.” |
| Interpretation | 20 | Give a cautious overall interpretation of results considering objectives, limitations, multiplicity of analyses, results from similar studies, and other relevant evidence | | Throughout Discussion. |
| Generalisability | 21 | Discuss the generalisability (external validity) of the study results | | Discussion, Paragraphs 2 & 3 |
| **Other information** | | | | |
| Funding | 22 | Give the source of funding and the role of the funders for the present study and, if applicable, for the original study on which the present article is based | | Following journal requirements, these have been submitted via the online submission forms to be published alongside the final published article and is included as a footnote. |

*Give information separately for cases and controls in case-control studies and, if applicable, for exposed and unexposed groups in cohort and cross-sectional studies.

Reference: Benchimol EI, Smeeth L, Guttmann A, Harron K, Moher D, Petersen I, Sørensen HT, von Elm E, Langan SM, the RECORD Working Committee. The REporting of studies Conducted using Observational Routinely-collected health Data (RECORD) Statement. PLoS Medicine 2015; in press. Checklist is protected under Creative Commons Attribution ([CC BY](http://creativecommons.org/licenses/by/4.0/)) license.
