# Supplementary material for: Hospital admission on weekends for patients who have surgery and 30-day mortality in Ontario, Canada: A matched cohort study
Source: PLoS Med. 2019 Jan 29;16(1):e1002731. doi: 10.1371/journal.pmed.1002731 (PMC6350956; doi:10.1371/journal.pmed.1002731)
Supplement: S1 Dataset Creation and Analysis Plan — (DOCX) [file pmed.1002731.s002.docx]

| General Description | |
| --- | --- |
| **Original project Name:** | The association between weekend admission and perioperative adverse outcomes for adults undergoing surgery: A matched cohort study |
| **Investigators:** | James O’Leary  Hannah Wunsch  Anne-Marie Leo  David Levin  Asad Siddiqui  Mark Crawford |
| **Objectives:** | The ***overall objective*** is to determine if adults undergoing noncardiac surgery have an increased risk of perioperative mortality if they are admitted to hospital or undergo surgery at the weekend compared with those individuals receiving the same care on weekdays.  The *specific objectives* of this project are to answer the following research questions: What is the risk of perioperative mortality for adults who undergo noncardiac surgery at the weekend compared with similar individuals who undergo similar surgery on weekdays?What is the risk of perioperative mortality for adults admitted to hospital at the weekend and subsequently undergo noncardiac surgery during the week compared with similar individuals who are admitted and undergo surgery on weekdays?What is the risk of perioperative mortality for elective and urgent weekend admissions compared with similar individuals who are admitted and undergo surgery on weekdays? |
| **ICES databases to be used:** | Ontario Registered Persons Database (RPDB)  Canadian Institutes for Health Information – Discharge Abstract Database (CIHI-DAD) |
| **Update History:** | 2016-10-14 DCP Creation  2016-11-02 Cohort definitions updated  2017-01-12 Matching updated and outcomes harmonized with objectives  2017-02-01 Matching updated and ICD-9 codes added  2017-10-10 Index dates clarified, outcomes updated, baseline characteristics clarified, matching ratio updated  2018-01-09 Cohort definition updated  2018-06-26 Objectives, covariates, and analysis updated in response to reviewers’ comments at PLOS Medicine |
| **Type of study:** | Retrospective matched cohort study |

| Cohorts | | | | |
| --- | --- | --- | --- | --- |
| **Exposed Cohort Definitions:** | General Description (N=212,387) | | | |
|  | *Step* | *Description* |  |  |
|  | 1 | All admissions between 1JAN2005 and 31DEC2015 where non-cardiac surgery was performed and indicated in any incode field [*Appendix]* (DAD).   - events NOT individuals - excluding admissions associated with: - obstetric interventions (5.^^.^^) - cardiovascular interventions (1.H^.^^.^^) - therapeutic interventions on the Great Vessels (1IA - 1IS) - therapeutic interventions on combined sites for Congenital Heart anomalies (1LA - 1LD | | |
|  | 2 | Age >= 18yr at time of surgery | | |
|  | 3 | We will identify 2 mutually exclusive exposed groups from the above cohort, those who:   1. were admitted to hospital at the weekend (Sat/Sun only) and the first eligible surgery was performed on the same weekend (Sat/Sun only) (identified using DAD Intervention Episode Start Date), or 2. were admitted at the weekend (Sat/Sun only) but whose first eligible surgery was performed on a following weekday (Monday to Thursday) only (identified using both DAD Admission Date and Time and Intervention Episode Start Date) | | |
| **Reference Cohort Definitions:** | General Description (N=1,153,834) | | | |
|  | *Step* | *Description* |  |  |
|  | 1 | All admissions between 1JAN2005 and 31DEC2015 where non-cardiac surgery was performed and indicated in any incode field [*Appendix]* (DAD).   - events NOT individuals - excluding admissions associated with: - obstetric interventions (5.^^.^^) - cardiovascular interventions (1.H^.^^.^^) - therapeutic interventions on the Great Vessels (1IA - 1IS) - therapeutic interventions on combined sites for Congenital Heart anomalies (1LA - 1LD) | | |
|  | 2 | Age >= 18yr at time of surgery | | |
|  | 3 | Adults who were admitted to hospital and underwent surgery on a weekday (Tuesday to Thursday) of the same week (identified using DAD Admission Date and Time and Intervention Episode Start Date | | |
| Variable Definitions | | | | |
| **Baseline Characteristics at index event:** | - Demographic characteristics: - Age category  1. Adult ≡ 18 to <50 yr 2. Older adult ≡ 50 to <65 yr 3. Geriatric adult ≡ ≥65 yr  - Sex - Income quintile - Year of treatment - Mode of admission (elective, urgent) - Institution - Teaching hospital (ICES defined) - Local health integration network (LHIN) - Rurality Index for Ontario - Resource Utilization Band - Length of stay for index event - Time interval between surgery and discharge date - Time interval between admission and surgery - OHIP SOB anesthesia base value (≤7 & >8) - Charlson co-morbidity index (using a 5-year look-back window) | | | |
| Matching | | | | |
| Adults in each exposure group (weekend admission with weekend or weekday surgery) will be matched (1:1) using a deterministic technique by variables to adjust for possible unmeasured confounding from age, healthcare utilization, time-trends, and socioeconomic status. | | | | |
| **Variables:** | *Item* | | *Descriptor* | |
|  | Time period when surgery was performed | | Calendar year | |
|  | Resource Utilization Band | | 0, nonusers; 1, healthy users; 2, users with low morbidity; 3, users with moderate morbidity; 4, users with high morbidity; and 5, users with very high morbidity | |
|  | Quintile of median neighborhood household income | |  | |
|  | Age at the time of the index event | | from Year of birth | |
|  | Rurality | | Rurality Index for Ontario | |
|  | Complexity of surgical procedure | | from incode1 field of DAD; physiological complexity defined using OHIP base values (≤7 & >8) | |
|  | Type of admission | | from DAD | |
| Outcome Definition(s) | | | | |
| **Primary Outcome:** | - All-cause 30-day mortality (from date of admission) (DAD, RPDB) | | | |
| **Secondary Outcome (for sensitivity analysis):** | - All-cause 30-day mortality (from date of surgery) (DAD, RPDB) | | | |
| Statistical Analysis | | | | |
| Descriptive statistics will be determined for participants according to pre-specified exposure groups (overall, day of surgery, urgency of admission) and their matched controls. The overall risk for outcomes with 95% confidence intervals (CIs) will be determined for the exposed and reference groups according to pre-specified subgroups of interest using matched pairs. To account for any correlation induced by matching, GEE-based multivariable logistic and linear regression analyses will be used.  Results will be summarized using odds ratio estimates and 95% CIs. Statistical significance will be defined as two-tailed P < 0.05.  Sensitivity analysis of 30-day all-cause mortality from date of hospital admission and from date of surgery: risk ratios for both outcome measures will be calculated to examine for a survivor bias.  **Additional Analysis to address reviewers’ comments from PLOS Medicine:**  1. Add Charlson Comorbidity, a mortality risk score (based on the Johns Hopkins Adjusted Clinical Group case-mix system), preoperative admission to a special care unit, and responsible surgical service to the covariates tested in models to provide more accurate clinical information on comorbidities, illness severity, and perioperative risk.  2. Models to test matched pairs nested within hospital clusters to account for assortative mixing of patients in hospitals.  3. Perform additional sensitivity analyses to test whether the increased time interval to surgery observed on weekends was contributing to differences between groups. Specifically, all analyses (overall and subgroup) to be performed adjusting for the time interval from admission to surgery in all models and, where appropriate, an interaction term between time to surgery and admission type (elective vs urgent) to be included. | | | | |

| **APPENDIX .** Noncardiac surgery definition | | |
| --- | --- | --- |
|  | **Category** | **CCI Code and Description** |
|  | All therapeutic interventions (this automatically excludes obstetric interventions) | 1.AA.* - 1.YZ.* |
|  | *Excluding:* | - Radiation interventions (1.^^.27.^^) - Brachytherapy interventions (1.^^.26.^^) - Therapeutic interventions of the gingiva (1.FD.^^.^^), tooth (1.FE.^^.^^), root of tooth (1.FF.^^.^^), oral and buccal mucosa (1.FG.^^.^^) not associated with operative anesthesia (CCI: 1.ZZ.11.^^) - Therapeutic interventions of the respiratory system (1.GZ.^^.^^) including replacement of endotracheal tube (1.GJ.50.CA-NG) - Management of internal device, gastrointestinal (1.NF.54.^^ or 1.NK.54.^^) - Implantation (1.NF.53.CA) or removal (1.NF.55.CA) of internal device (per-orifice), stomach - Therapeutic Interventions on the Digestive System NEC (1.OZ.^^.^^) - Pharmacotherapy interventions (1..^^.35.^^) - Drainage interventions (1.^^.52.^^), with the exception of 1.AA.52.^^ (drainage of meninges and dura mater of brain) - Therapeutic interventions of the skin (1.Y^.12.^^) - Dialysis interventions (1.^^.21) - Immobilization interventions (1.^^.03.^^) not associated with operative anesthesia (1.ZZ.11.^^) - Dressing interventions (1.^^.14.^^) not associated with operative anesthesia (CCI: 1.ZZ.11.^^) - Exercise (CCI: 1..^^.02.^^), mobilization (1..^^.04.^^), manipulation (CCI: 1..^^.05.^^), hypothermy (1..^^.06.^^), hyperthermy (1..^^.07.^^), therapy not otherwise specified (1..^^.12.^^) - Insertion of simple intravenous line (1.KX.53.^^) - Stimulation, peripheral nerves (1.BX.09.^^) - Procurement procedures from deceased donor (1.^^.58.^^-XX-K) |
