# Supplementary material for: Hospital admission on weekends for patients who have surgery and 30-day mortality in Ontario, Canada: A matched cohort study
Source: PLoS Med. 2019 Jan 29;16(1):e1002731. doi: 10.1371/journal.pmed.1002731 (PMC6350956; doi:10.1371/journal.pmed.1002731)
Supplement: S1 Table — (DOCX) [file pmed.1002731.s003.docx]

**S1 Table.** Surgical procedure definition.

| Surgical procedures were identified using Canadian Classification of Health Interventions (CCI) therapeutic intervention codes. Surgical procedures defined by CCI therapeutic intervention categories 1.AA.^^.^^ - 1.YZ.^^.^^ in any incode field of the Discharge Abstract Database (DAD) were eligible for inclusion, except the following therapeutic interventions: |
| --- |
| - Brachytherapy interventions (1.^^.26.^^) - Cardiovascular interventions (1.H^.^^.^^) - Dialysis interventions (1.^^.21) - Drainage interventions (1.^^.52.^^), with the exception of 1.AA.52.^^ (drainage of meninges and dura mater of brain) - Dressing interventions (1.^^.14.^^) not associated with operative anesthesia (1.ZZ.11.^^) - Management of internal device, gastrointestinal (1.NF.54.^^ or 1.NK.54.^^) - Exercise (1..^^.02.^^) - Hypothermy (1..^^.06.^^) - Hyperthermy (1..^^.07.^^) - Immobilization interventions (1.^^.03.^^) not associated with operative anesthesia (1.ZZ.11.^^) - Implantation (1.NF.53.CA) or removal (1.NF.55.CA) of internal device (per-orifice), stomach - Insertion of simple intravenous line (1.KX.53.^^) - Mobilization (1..^^.04.^^) - Manipulation (1..^^.05.^^) - Pharmacotherapy interventions (1..^^.35.^^) - Procurement procedures from deceased donor (1.^^.58.^^-XX-K) - Radiation interventions (1.^^.27.^^) - Stimulation, peripheral nerves (1.BX.09.^^) - Therapeutic interventions on combined sites for Congenital Heart anomalies (1LA.^^.^^ - 1LD.^^.^^) - Therapeutic Interventions on the Digestive System NEC (1.OZ.^^.^^) - Therapeutic interventions of the gingiva (1.FD.^^.^^), tooth (1.FE.^^.^^), root of tooth (1.FF.^^.^^), oral and buccal mucosa (1.FG.^^.^^) not associated with operative anesthesia (1.ZZ.11.^^) - Therapeutic interventions on the Great Vessels (1IA.^^.^^ - 1IS.^^.^^) - Therapeutic interventions of the respiratory system (1.GZ.^^.^^) including replacement of endotracheal tube (1.GJ.50.CA-NG) - Therapeutic interventions of the skin (1.Y^.12.^^) - Therapy not otherwise specified (1..^^.12.^^) |
