# Supplementary material for: Hospital admission on weekends for patients who have surgery and 30-day mortality in Ontario, Canada: A matched cohort study
Source: PLoS Med. 2019 Jan 29;16(1):e1002731. doi: 10.1371/journal.pmed.1002731 (PMC6350956; doi:10.1371/journal.pmed.1002731)
Supplement: S2 Table — (DOCX) [file pmed.1002731.s004.docx]

**S2 Table.** Characteristics of all adult admissions with noncardiac surgery performed in Ontario hospitals between January 2005 and December 2015, classified by day (weekend or weekday) of admission and surgery.

| **Characteristic** | **All admissions** | **Weekend admission and surgery** | **Weekend admission and weekday surgery** | **Weekday admission and surgery** |
| --- | --- | --- | --- | --- |
|  | **N = 1,366,221** | **n = 115,936** | **n = 96,451** | **n = 1,153,834** |
| Age category, *n(%)*  18 to 49 yr  50 to 64 yr  ≥65 yr | 409,631 (30.0)  398,596 (29.2)  557,994 (40.8) | 50,386 (43.5)  25,897 (22.3)  39,653 (34.2) | 26,420 (27.4)  24,058 (24.9)  45,973 (47.7) | 332,825 (28.8)  348,641 (30.2)  472,368 (40.9) |
| Male, *n(%)* | 614,839 (45.0) | 57,964 (50.0) | 51,686 (53.6) | 505,189 (43.8) |
| Median neighborhood income quintile, *n(%)*  Missing  1 - Lowest  2  3  4  5 | 6,595 (0.5)  255,978 (18.7)  270,438 (19.8)  271,530 (19.9)  281,137 (20.6)  280,543 (20.5) | 729 (0.6)  24,209 (20.9)  23,323 (20.1)  22,757 (19.6)  23,098 (19.9)  21,820 (18.8) | 600 (0.6)  19,761 (20.5)  18,945 (19.6)  18,731 (19.4)  19,373 (20.1)  19,041 (19.7) | 5,266 (0.5)  212,008 (18.4)  228,170 (19.8)  230,042 (19.9)  238,666 (20.7)  239,682 (20.8) |
| Rural home Location, *n(%)* | 194,379 (14.2) | 14,774 (12.7) | 12,223 (12.7) | 167,382 (14.5) |
| Resource utilization band^a^, *n(%)*  0 - Lowest  1  2  3  4  5 | 513 (0.0)  1,146 (0.1)  47,329 (3.5)  530,479 (38.8)  409,983 (30.0)  376,771 (27.6) | 134 (0.1)  290 (0.3)  10,387 (9.0)  40,568 (35.0)  31,318 (27.0)  33,239 (28.7) | 14 (0.0)  39 (0.0)  4,739 (4.9)  28,832 (29.9)  24,736 (25.6)  38,091 (39.5) | 365 (0.0)  817 (0.1)  32,203 (2.8)  461,079 (40.0)  353,929 (30.7)  305,441 (26.5) |
| Charlson Comorbidity Index, *n*(%)  0  1  ≥2 | 1,043,664 (76.4)  104,436 (7.6)  218,121 (16.0) | 92,198 (79.5)  8,471 (7.3)  15,267 (13.2) | 65,861 (68.3)  8,877 (9.2)  21,713 (22.5) | 885,605 (76.8)  87,088 (7.5)  181,141 (15.7) |
| Mortality Risk Score^b^, *mean ± SD* | 72.25 ± 23.01 | 68.65 ± 27.28 | 78.42 ± 26.71 | 72.10 ± 22.09 |
| Year of admission, *n(%)*  2005  2006  2007  2008  2009  2010  2011  2012  2013  2014  2015 | 125,729 (9.2)  125,266 (9.2)  124,892 (9.1)  123,387 (9.0)  122,056 (8.9)  120,734 (8.8)  123,069 (9.0)  124,915 (9.1)  124,891 (9.1)  125,521 (9.2)  125,761 (9.2) | 10,286 (8.9)  10,199 (8.8)  10,483 (9.0)  10,363 (8.9)  10,274 (8.9)  10,400 (9.0)  10,386 (9.0)  10,722 (9.2)  10,974 (9.5)  11,066 (9.5)  10,783 (9.3) | 8,382 (8.7)  8,310 (8.6)  8,359 (8.7)  8,388 (8.7)  8,724 (9.0)  8,546 (8.9)  8,759 (9.1)  9,010 (9.3)  9,060 (9.4)  9,573 (9.9)  9,340 (9.7) | 107,061 (9.3)  106,757 (9.3)  106,050 (9.2)  104,636 (9.1)  103,058 (8.9)  101,788 (8.8)  103,924 (9.0)  105,183 (9.1)  104,857 (9.1)  104,882 (9.1)  105,638 (9.2) |
| Elective admission*, n(%)* | 977,596 (71.6) | 7,344 (6.3) | 21,649 (22.4) | 948,603 (82.2) |
| Admission to a teaching hospital, *n(%)* | 501,899 (36.7) | 36,190 (31.2) | 34,237 (35.5) | 431,472 (37.4) |
| Surgical procedures with ≥8 OHIP anesthesia basic units, *n(%)* | 333,769 (24.4) | 14,780 (12.7) | 14,455 (15.0) | 304,534 (26.4) |
| Admitted to a special care unit prior to surgery, *n(%)* | 129,324 (9.5%) | 14,097 (12.2%) | 12,120 (12.6%) | 103,107 (8.9%) |
| Days from admission to surgery, *mean ± SD* | 0.3 ± 0.7 | 0.2 ± 0.4 | 2.0 ± 1.2 | 0.1 ± 0.4 |
| Length of hospital stay, *mean ± SD* | 4.4 ± 7.3 | 5.5 ± 11.1 | 8.3 ± 11.9 | 3.9 ± 6.1 |

SD, standard deviation; OHIP, Ontario Health Insurance Plan; S, suppressed percentage (cell counts <6 cannot be reported)

^a^Resource utilization band is a ranking system of overall morbidity based on the Johns Hopkins Adjusted Clinical Group case-mix system

^b^Mortality Risk Score based on the Johns Hopkins Adjusted Clinical Group case-mix system
