# Supplementary material for: Hospital admission on weekends for patients who have surgery and 30-day mortality in Ontario, Canada: A matched cohort study
Source: PLoS Med. 2019 Jan 29;16(1):e1002731. doi: 10.1371/journal.pmed.1002731 (PMC6350956; doi:10.1371/journal.pmed.1002731)
Supplement: S3 Table — (DOCX) [file pmed.1002731.s005.docx]

**S3 Table.** Characteristics of matched and unmatched weekend admissions with noncardiac surgery performed in Ontario hospitals between January 2005 and December 2015.

| **Characteristic** | **Weekend admissions in unmatched cohort** | **Weekend admissions in matched cohort** |  |
| --- | --- | --- | --- |
|  | **n = 212,387** | **n = 159,101** | **P**^a^ |
| Age category, *n(%)*  18 to 49 yr  50 to 64 yr  ≥65 yr | 76,806 (36.2)  49,955 (23.5)  85,626 (40.3) | 57,869 (36.4)  38,242 (24.0)  62,990 (39.6) | <0.001 |
| Male, *n(%)* | 109,650 (51.6) | 81,273 (51.1) | 0.001 |
| Median neighborhood income quintile, *n(%)*  Missing  1 - Lowest  2  3  4  5 | 1,329 (0.6)  43,970 (20.7)  42,268 (19.9)  41,488 (19.5)  42,471 (20.0)  40,861 (19.2) | 99 (0.1)  33,016 (20.8)  31,881 (20.0)  31,157 (19.6)  32,146 (20.2)  30,802 (19.4) | <0.001 |
| Rural home Location, *n(%)* | 26,997 (12.7) | 11,922 (7.5) | <0.001 |
| Resource utilization band^b^, *n(%)*  0 - Lowest  1  2  3  4  5 | 146-150 (S)  327-331 (S)  15,126 (7.1)  69,400 (32.7)  56,054 (26.4)  71,330 (33.6) | ≤5 (S)  8-12 (S)  9,250 (5.8)  56,420 (35.5)  41,564 (26.1)  51,855 (32.6) | <0.001 |
| Charlson Comorbidity Index, *n*(%)  0  1  ≥2 | 158,059 (74.4)  17,348 (8.2)  36,980 (17.4) | 121,124 (76.1)  12,366 (7.8)  25,611 (16.1) | <0.001 |
| Mortality Risk Score^c^, *mean ± SD* |  | 72.43 ± 27.45 |  |
| Year of admission, *n(%)*  2005  2006  2007  2008  2009  2010  2011  2012  2013  2014  2015 | 18,668 (8.8)  18,509 (8.7)  18,842 (8.9)  18,751 (8.8)  18,998 (8.9)  18,946 (8.9)  19,145 (9.0)  19,732 (9.3)  20,034 (9.4)  20,639 (9.7)  20,123 (9.5) | 14,195 (8.9)  13,905 (8.7)  13,975 (8.8)  14,108 (8.9)  14,213 (8.9)  14,017 (8.8)  14,416 (9.1)  14,764 (9.3)  14,968 (9.4)  15,422 (9.7)  15,118 (9.5) | 0.9 |
| Elective admission*, n(%)* | 28,994 (13.7) | 25,872 (16.3) | <.001 |
| Admission to a teaching hospital, *n(%)* | 70,427 (33.2) | 47,780 (30.0) | <.001 |
| Surgical procedures with ≥8 OHIP anesthesia basic units, *n(%)* | 29,235 (13.8) | 14,007 (8.8) | <.001 |
| Admitted to a special care unit prior to surgery, *n(%)* |  |  |  |
| Days from admission to surgery, *mean ± SD* | 1.0 ± 1.3 | 1.0 ± 1.2 | 0.002 |
| Length of hospital stay, *mean ± SD* | 6.8 ± 11.6 | 6.5 ± 10.8 | <0.001 |

SD, standard deviation; OHIP, Ontario Health Insurance Plan; S, suppressed percentage (cell counts <6 cannot be reported)

^a^P values not reported for variables used in exacting matching of study groups

^b^Resource utilization band is a ranking system of overall morbidity based on the Johns Hopkins Adjusted Clinical Group case-mix system

^c^Mortality Risk Score based on the Johns Hopkins Adjusted Clinical Group case-mix system
