# Supplementary material for: Hospital admission on weekends for patients who have surgery and 30-day mortality in Ontario, Canada: A matched cohort study
Source: PLoS Med. 2019 Jan 29;16(1):e1002731. doi: 10.1371/journal.pmed.1002731 (PMC6350956; doi:10.1371/journal.pmed.1002731)
Supplement: S5 Table — (DOCX) [file pmed.1002731.s007.docx]

**S5 Table.** Characteristics of eligible weekend admissions with weekday noncardiac surgery performed in Ontario hospitals between January 2005 and December 2015 matched directly to weekday admissions on age, complexity of surgical procedure, median neighborhood household income, resource utilization band, year of admission, and urgency of admission.

| **Characteristic** | **Weekend admission with weekday surgery** | **Weekday admission and surgery** |  |
| --- | --- | --- | --- |
|  | **n = 73,357** | **n = 73,357** | **P**^a^ |
| Age category, *n(%)*  18 to 49 yr  50 to 64 yr  ≥65 yr | 20,546 (28.0)  18,882 (25.7)  33,929 (46.3) | 20,531 (28.0)  18,901 (25.8)  33,925 (46.2) | NA |
| Male, *n(%)* | 39,875 (54.4) | 32,089 (43.7) | <0.001 |
| Median neighborhood income quintile, *n(%)*  Missing  1 - Lowest  2  3  4  5 | 56 (0.1)  15,055 (20.5)  14,389 (19.6)  14,188 (19.3)  14,940 (20.4)  14,729 (20.1) | 56 (0.1)  15,055 (20.5)  14,389 (19.6)  14,188 (19.3)  14,940 (20.4)  14,729 (20.1) | NA |
| Rural home Location, *n(%)* | 5,658 (7.7) | 5,658 (7.7) | NA |
| Resource utilization band^b^, *n(%)*  0 - Lowest  1  2  3  4  5 | 0 (0.0)  0 (0.0)  3,128 (4.3)  24,250 (33.1)  18,365 (25.0)  27,614 (37.6) | 0 (0.0)  0 (0.0)  3,128 (4.3)  24,250 (33.1)  18,365 (25.0)  27,614 (37.6) | NA |
| Charlson Comorbidity Index, *n*(%)  0  1  ≥2 | 51,972 (70.8)  6,360 (8.7)  15,025 (20.5) | 53,794 (73.3)  6,187 (8.4)  13,376 (18.2) | <0.001 |
| Mortality Risk Score^c^, *mean ± SD* |  |  |  |
| Year of admission, *n(%)*  2005  2006  2007  2008  2009  2010  2011  2012  2013  2014  2015 | 6,473 (8.8)  6,420 (8.8)  6,360 (8.7)  6,449 (8.8)  6,651 (9.1)  6,421 (8.8)  6,654 (9.1)  6,886 (9.4)  6,814 (9.3)  7,194 (9.8)  7,035 (9.6) | 6,473 (8.8)  6,420 (8.8)  6,360 (8.7)  6,449 (8.8)  6,651 (9.1)  6,421 (8.8)  6,654 (9.1)  6,886 (9.4)  6,814 (9.3)  7,194 (9.8)  7,035 (9.6) | NA |
| Elective admission*, n(%)* | 19,467 (26.5) | 19,467 (26.5) | NA |
| Admission to a teaching hospital, *n(%)* | 23,275 (31.7) | 23,223 (31.7) | 0.77 |
| Surgical procedures with ≥8 OHIP anesthesia basic units, *n(%)* | 7,232 (9.9) | 7,232 (9.9) | NA |
| Admitted to a special care unit prior to surgery, *n(%)* |  |  |  |
| Days from admission to surgery, *mean ± SD* | 2.0 ± 1.2 | 0.4 ± 0.6 | <.001 |
| Length of hospital stay, *mean ± SD* | 7.9 ± 11.4 | 6.0 ± 12.0 | <.001 |

SD, standard deviation; OHIP, Ontario Health Insurance Plan; S, suppressed percentage (cell counts <6 cannot be reported)

^a^P values not reported for variables used in exacting matching of study groups

^b^Resource utilization band is a ranking system of overall morbidity based on the Johns Hopkins Adjusted Clinical Group case-mix system

^c^Mortality Risk Score based on the Johns Hopkins Adjusted Clinical Group case-mix system
