# Supplementary material for: Hospital admission on weekends for patients who have surgery and 30-day mortality in Ontario, Canada: A matched cohort study
Source: PLoS Med. 2019 Jan 29;16(1):e1002731. doi: 10.1371/journal.pmed.1002731 (PMC6350956; doi:10.1371/journal.pmed.1002731)
Supplement: S6 Table — (DOCX) [file pmed.1002731.s008.docx]

**S6 Table**. Frequency of the 10 most common noncardiac surgical procedures^a^ performed for weekend and weekday admissions in the matched cohort, classified by day of surgery for weekend admissions (weekday or weekend), type of admission (elective or urgent), and sex.

|  | **Weekend admission** | **Weekday admission** |
| --- | --- | --- |
| **All weekend admissions with weekend surgery (n = 85,744)** |  |  |
| *Male* |  |  |
| Excision total, appendix using endoscopic [laparoscopic] approach | 9,165 | 8,962 |
| Excision total, gallbladder endoscopic [laparoscopic] approach without extraction (of calculi) cholecystectomy alone | 2,179 | 1,804 |
| Fixation, ankle joint open approach with bone autograft using screw, plate and screw | 2,313 | 1,647 |
| Fixation, femur open approach fixation device alone using intramedullary nail | 1,933 | 1,671 |
| Repair, muscles of the chest and abdomen open approach without tissue [e.g. suturing or stapling] | 1,640 | 1,646 |
| Fixation, tibia and fibula open approach fixation device alone using intramedullary nail | 1,698 | 1,295 |
| Excision partial, large intestine open approach Enterocolostomy anastomosis technique | 1,077 | 1,407 |
| Implantation of internal device, hip joint open approach uncemented single-component prosthetic device [femoral] | 1,150 | 1,119 |
| Fixation, hip joint open approach fixation device alone using plate, screw | 1,107 | 1,046 |
| Destruction, ureter endoscopic per orifice approach using laser | 883 | 788 |
| *Female* |  |  |
| Excision total, appendix using endoscopic [laparoscopic] approach | 9,333 | 8,558 |
| Fixation, femur open approach fixation device alone using intramedullary nail | 3,925 | 3,545 |
| Excision total, gallbladder endoscopic [laparoscopic] approach without extraction (of calculi) cholecystectomy alone | 3,722 | 3,441 |
| Implantation of internal device, hip joint open approach uncemented dual-component prosthetic device [femoral & acetabular] | 2,872 | 2,609 |
| Fixation, ankle joint open approach fixation device alone using screw, plate and screw | 2,889 | 2,319 |
| Fixation, hip joint open approach fixation device alone using plate, screw | 2,463 | 2,127 |
| Excision partial, large intestine endoscopic [laparoscopic, laparoscopic-assisted, hand-assisted] approach enterocolostomy anastomosis technique | 1,278 | 1,365 |
| Repair, muscles of the chest and abdomen open approach without tissue [e.g. suturing or stapling] | 1,316 | 1,232 |
| Fixation, radius and ulna open approach no tissue used using plate, screw | 1,187 | . |
| Fixation, tibia and fibula open approach fixation device alone using intramedullary nail | 1,054 | . |
| Implantation of internal device, knee joint with synthetic material (e.g. bone paste, cement, Dynagraft, Osteoset) using cement spacer (temporary) [impregnated with antibiotics] | . | 990 |
| Excision total, uterus and surrounding structures using open approach | . | 866 |
| **All weekend admissions with weekday surgery (n = 73,357)** |  |  |
| *Male* |  |  |
| Repair, muscles of the chest and abdomen open approach using special excisional technique | 12,709 | 3,088 |
| Excision total, appendix using endoscopic [laparoscopic] approach | 894 | 3,379 |
| Excision partial, large intestine open approach Enterocolostomy anastomosis technique | 2,102 | 1,675 |
| Excision total, gallbladder endoscopic [laparoscopic] approach without extraction (of calculi) cholecystectomy alone | 1,922 | 1,226 |
| Fixation, femur open approach fixation device alone using screw, plate and screw | 1,542 | 1,402 |
| Implantation of internal device, hip joint open approach uncemented dual-component prosthetic device [femoral & acetabular] | 1,269 | 1,166 |
| Fixation, ankle joint open approach fixation device alone using screw, plate and screw | 1,084 | 979 |
| Excision partial, prostate, endoscopic per orifice approach (TURP) using device NEC | . | 1,905 |
| Fixation, hip joint open approach fixation device alone using plate, screw | 892 | 897 |
| Fixation, tibia and fibula open approach fixation device alone using pin, nail | 931 | 743 |
| Extraction, bile ducts endoscopic [retrograde] per orifice approach [ERC] using balloon device | 1,434 | . |
| *Female* |  |  |
| Fixation, femur open approach fixation device alone using intramedullary nail | 3,588 | 3,322 |
| Implantation of internal device, hip joint open approach with synthetic material (e.g. bone paste, cement, Dynagraft, Osteoset) using dual-component prosthetic device [femoral & acetabular] | 2,985 | 2,720 |
| Excision total, gallbladder endoscopic [laparoscopic] approach without extraction (of calculi) cholecystectomy alone | 3,389 | 2,082 |
| Excision total, appendix using endoscopic [laparoscopic] approach | 1,077 | 3,716 |
| Excision total, uterus and surrounding structures using combined laparoscopic and vaginal approach | . | 4,660 |
| Fixation, hip joint open approach with bone homograft using plate, screw | 2,002 | 2,060 |
| Excision partial, large intestine open approach Enterocolostomy anastomosis technique | 1,987 | 1,807 |
| Fixation, ankle joint open approach fixation device alone using screw, plate and screw | 1,533 | 1,532 |
| Repair, muscles of the chest and abdomen open approach using special excisional technique | 1,561 | 1,486 |
| Extraction, bile ducts endoscopic [retrograde] per orifice approach [ERC] using basket [dormia] device | 1,969 | . |
| Excision partial, uterus and surrounding structures endoscopic [laparoscopic] approach using device NEC | . | 899 |
| Dilation, bile ducts endoscopic [retrograde] per orifice approach [ERC] using incision alone | 878 | . |
| **Urgent weekend admissions with weekend surgery (n = 79,339)** |  |  |
| *Male* |  |  |
| Excision total, appendix using endoscopic [laparoscopic] approach | 9,116 | 8,959 |
| Excision total, gallbladder endoscopic [laparoscopic] approach without extraction (of calculi) cholecystectomy alone | 2,134 | 1,784 |
| Fixation, ankle joint open approach with bone autograft using screw, plate and screw | 2,096 | 1,622 |
| Fixation, femur open approach fixation device alone using intramedullary nail | 1,884 | 1,665 |
| Repair, muscles of the chest and abdomen open approach without tissue [e.g. suturing or stapling] | 1,588 | 1,460 |
| Fixation, tibia and fibula open approach fixation device alone using intramedullary nail | 1,593 | 1,276 |
| Excision partial, large intestine open approach Enterocolostomy anastomosis technique | 993 | 1,329 |
| Fixation, hip joint open approach fixation device alone using plate, screw | 1,069 | 1,043 |
| Implantation of internal device, hip joint open approach with synthetic material (e.g. bone paste, cement, Dynagraft, Osteoset) using single-component prosthetic device [femoral] | 1,001 | 968 |
| Destruction, ureter endoscopic per orifice approach using laser | 833 | 784 |
| *Female* |  |  |
| Excision total, appendix using endoscopic [laparoscopic] approach | 9,264 | 8,555 |
| Fixation, femur open approach fixation device alone using intramedullary nail | 3831 | 3533 |
| Excision total, gallbladder endoscopic [laparoscopic] approach without extraction (of calculi) cholecystectomy alone | 3647 | 3416 |
| Implantation of internal device, hip joint open approach uncemented dual-component prosthetic device [femoral & acetabular] | 2649 | 2416 |
| Fixation, ankle joint open approach fixation device alone using screw, plate and screw | 2640 | 2298 |
| Fixation, hip joint open approach fixation device alone using plate, screw | 2387 | 2127 |
| Excision partial, large intestine endoscopic [laparoscopic, laparoscopic-assisted, hand-assisted] approach enterocolostomy anastomosis technique | 1175 | 1291 |
| Repair, muscles of the chest and abdomen open approach without tissue [e.g. suturing or stapling] | 1278 | 1146 |
| Fixation, tibia and fibula open approach fixation device alone using intramedullary nail | 978 | 833 |
| Fixation, radius and ulna open approach no tissue used using plate, screw | 1021 | . |
| Extraction, bile ducts endoscopic [retrograde] per orifice approach [ERC] using balloon device | . | 777 |
| **Urgent weekend admissions with weekday surgery (n = 53,890)** |  |  |
| *Male* |  |  |
| Excision total, appendix using endoscopic [laparoscopic] approach | 889 | 3,341 |
| Excision partial, large intestine open approach Enterocolostomy anastomosis technique | 1,815 | 1,206 |
| Excision total, gallbladder endoscopic [laparoscopic] approach without extraction (of calculi) cholecystectomy alone | 1,885 | 1,110 |
| Fixation, femur open approach fixation device alone using screw, plate and screw | 1,479 | 1,399 |
| Implantation of internal device, hip joint open approach uncemented dual-component prosthetic device [femoral & acetabular] | 1,151 | 981 |
| Fixation, ankle joint open approach fixation device alone using screw, plate and screw | 1,036 | 967 |
| Fixation, hip joint open approach fixation device alone using plate, screw | 861 | 895 |
| Fixation, tibia and fibula open approach fixation device alone using pin, nail | 883 | 732 |
| Extraction, bile ducts endoscopic [retrograde] per orifice approach [ERC] using balloon device | 1,421 | . |
| Repair, muscles of the chest and abdomen open approach using synthetic tissue [e.g. mesh, sponge] | . | 1,133 |
| Dilation, bile ducts endoscopic [retrograde] per orifice approach [e.g. ERC] using rigid dilator [e.g. stent] | 726 | . |
| *Female* |  |  |
| Fixation, femur open approach fixation device alone using intramedullary nail | 3,476 | 3,316 |
| Excision total, gallbladder endoscopic [laparoscopic] approach without extraction (of calculi) cholecystectomy alone | 3,333 | 1,924 |
| Implantation of internal device, hip joint open approach with synthetic material (e.g. bone paste, cement, Dynagraft, Osteoset) using dual-component prosthetic device [femoral & acetabular] | 2,770 | 2,456 |
| Excision total, appendix using endoscopic [laparoscopic] approach | 1,067 | 3,684 |
| Fixation, hip joint open approach fixation device alone using plate, screw | 1,931 | 2,058 |
| Fixation, ankle joint open approach fixation device alone using screw, plate and screw | 1,475 | 1,524 |
| Excision partial, large intestine open approach Enterocolostomy anastomosis technique | 1,661 | 1,309 |
| Extraction, bile ducts endoscopic [retrograde] per orifice approach [ERC] using basket [dormia] device | 1,954 | 598 |
| Repair, muscles of the chest and abdomen endoscopic [laparoscopic] approach without tissue [e.g. suturing or stapling] | . | 975 |
| Dilation, bile ducts endoscopic [retrograde] per orifice approach [ERC] using incision alone | 865 | . |
| Fixation, tibia and fibula open approach fixation device alone using intramedullary nail | 654 | . |
| Excision partial, small intestine endoscopic [laparoscopic] approach Enteroenterostomy anastomosis technique | . | 620 |
| **Elective weekend admissions with weekend surgery (n = 6,405)** |  |  |
| *Male* |  |  |
| Implantation of internal device, knee joint with synthetic material | 187 | 551 |
| Excision partial, prostate endoscopic per orifice approach [transurethral] using device NEC | 212 | 227 |
| Implantation of internal device, hip joint open approach uncemented single-component prosthetic device [femoral] | 149 | 151 |
| Fixation, ankle joint open approach fixation device alone using screw, plate and screw | 217 | . |
| Repair, muscles of the chest and abdomen open approach using special excisional technique | . | 186 |
| Excision partial, large intestine open approach Enterocolostomy anastomosis technique | 84 | 78 |
| Excision partial, bladder using endoscopic per orifice approach | . | 150 |
| Transplant, kidney using deceased donor kidney | 114 | . |
| Fixation, radius and ulna open approach no tissue used using plate, screw | 110 | . |
| Fixation, tibia and fibula open approach fixation device alone using plate, screw | 105 | . |
| Excision partial, rectum open abdominal approach [e.g. anterior] stoma formation with distal closure | . | 77 |
| Repair, tendons of lower leg [around knee] using apposition technique [tendon sutured to tendon] with autograft [e.g. tendon, fascia] | 71 | . |
| Repair, tendons of ankle and foot using tenodesis with screw fixation [ tendon with a bone plug fixed to bone with screw] simple repair (without graft or transfer) | 68 | . |
| Extraction, carotid artery open approach using autograft using device NEC | . | 47 |
| Repair by decreasing size, uvula using open approach | . | 43 |
| Excision total, tonsils and adenoids tonsillectomy alone using device NEC | . | 33 |
| *Female* |  |  |
| Implantation of internal device, knee joint with synthetic material (e.g. bone paste, cement, Dynagraft, Osteoset) using dual component prosthetic device | 245 | 866 |
| Excision total, uterus and surrounding structures using vaginal approach | 161 | 471 |
| Implantation of internal device, hip joint open approach using bone autograft [uncemented] dual-component prosthetic device [femoral & acetabular] | 223 | 193 |
| Fixation, ankle joint open approach fixation device alone using screw, plate and screw | 249 | . |
| Excision partial, large intestine endoscopic [laparoscopic, laparoscopic-assisted, hand-assisted] approach colocolostomy anastomosis technique | 103 | 74 |
| Fixation, radius and ulna open approach no tissue used using plate, screw | 166 | . |
| Repair by decreasing size, stomach endoscopic [laparoscopic] approach using gastric bypass technique with gastroenterostomy [e.g. Roux-en-Y] | 92 | 67 |
| Fixation, humerus open approach fixation device alone using plate, screw | 114 | . |
| Fixation, bladder neck combined per orifice (vaginal) and open (abdominal) approach using autograft (e.g. fascia lata sling, rectus fascia) | . | 104 |
| Fixation, femur open approach fixation device alone using screw, plate and screw | 94 | . |
| Repair, muscles of the chest and abdomen open approach without tissue [e.g. suturing or stapling] | . | 86 |
| Excision partial, rectum open abdominal [e.g. anterior] approach colorectal anastomosis technique | . | 80 |
| Fixation, hip joint open approach fixation device alone using plate, screw | 76 | . |
| Excision partial, uterus and surrounding structures open approach using device NEC | . | 73 |
| Repair by decreasing size, breast using simple excisional technique with local flap [e.g. inferior, vertical or central] | . | 67 |
| **Elective weekend admissions with weekday surgery (n = 19,467)** |  |  |
| *Male* |  |  |
| Repair, muscles of the chest and abdomen open approach using special excisional technique | 12,332 | 1,955 |
| Excision partial, prostate endoscopic per orifice approach [transurethral] using device NEC | . | 1,523 |
| Excision partial, large intestine endoscopic [laparoscopic, laparoscopic-assisted, hand-assisted] approach enterocolostomy anastomosis technique | 287 | 469 |
| Implantation of internal device, knee joint with combined sources of tissue | . | 414 |
| Implantation of internal device, hip joint open approach uncemented dual-component prosthetic device [femoral & acetabular] | 118 | 185 |
| Repair, cruciate ligaments of knee endoscopic (and open) approach with meniscectomy or meniscoplasty using autograft [e.g. tendon with bone plug] and biodegradable device [e.g. biostinger, fastener, anchor, arrow, LactoSorb plate, staple, dart] | . | 173 |
| Repair, abdominal aorta using open approach with synthetic material [e.g. Teflon felt, Dacron, Nylon, Orlon] | 71 | 83 |
| Excision partial, bladder using open approach | . | 134 |
| Excision total, gallbladder open approach without extraction of calculi cholecystectomy alone | . | 116 |
| Transfusion, bone marrow infusion of autologous bone marrow | 113 | . |
| Bypass, arteries of leg NEC using autograft [e.g. saphenous vein] bypass terminating in lower limb vein(e.g. femoral artery to saphenous vein for long term hemodialysis) | 101 | . |
| Implantation of internal device, stomach of (gastric) tube using percutaneous approach | 89 | . |
| Excision total, rectum abdominal [anterior] approach pouch formation | . | 84 |
| Excision partial, soft tissue of leg using simple apposition technique [e.g. suture, staple] (for closure of surgical defect) | 84 | . |
| Excision partial, rectum open abdominal approach [e.g. anterior] stoma formation with distal closure | 82 | . |
| Implantation of internal device, small intestine of feeding tube [jejunal] using per orifice approach [e.g. naso intestinal] | 68 | . |
| *Female* |  |  |
| Excision total, uterus and surrounding structures using open approach | 97 | 4,446 |
| Repair, muscles of the chest and abdomen open approach using special excisional technique | 1,132 | 511 |
| Excision partial, large intestine endoscopic per orifice approach Simple excisional technique | 326 | 498 |
| Excision partial, uterus and surrounding structures endoscopic [laparoscopic] approach using device NEC | . | 677 |
| Implantation of internal device, knee joint with synthetic material | 58 | 602 |
| Repair, vagina endoscopic [laparoscopic] approach using synthetic material | . | 554 |
| Repair by decreasing size, breast using simple excisional technique with local flap [e.g. inferior, vertical or central] | . | 471 |
| Excision total, ovary with fallopian tube using open approach | . | 379 |
| Excision (modified) radical, breast without tissue | . | 289 |
| Excision partial, breast using open approach with simple apposition of tissue (e.g. suturing) | . | 276 |
| Implantation of internal device, hip joint using combined sources of tissue (e.g. bone graft, cement, paste) using dual-component prosthetic device [femoral & acetabular] | 215 | . |
| Fixation, femur open approach fixation device alone using intramedullary nail | 112 | . |
| Excision partial, rectum endoscopic [laparoscopic, laparoscopic-assisted, hand-assisted] approach colorectal anastomosis technique | 77 | . |
| Excision partial, soft tissue of leg using simple apposition technique [e.g. suture, staple] (for closure of surgical defect) | 77 | . |
| Transfusion, bone marrow infusion of autologous bone marrow | 73 | . |
| Fixation, hip joint open approach with bone homograft using plate, screw | 71 | . |

^a^Procedures are described using Canadian Classification of Health Interventions codes, available at https://www.cihi.ca/en/cci_vol3_2009_en.pdf
