# Supplementary material for: Hospital admission on weekends for patients who have surgery and 30-day mortality in Ontario, Canada: A matched cohort study
Source: PLoS Med. 2019 Jan 29;16(1):e1002731. doi: 10.1371/journal.pmed.1002731 (PMC6350956; doi:10.1371/journal.pmed.1002731)
Supplement: S7 Table — (DOCX) [file pmed.1002731.s009.docx]

**S7 Table.** Characteristics of elective admissions included in the matched cohorts, classified by day of surgery (weekend or weekday).

| **Characteristic** | **Weekend elective admission and surgery** | **Weekday admission and surgery** |  | **Weekend elective admission and weekday surgery** | **Weekday admission and surgery** |  |
| --- | --- | --- | --- | --- | --- | --- |
|  | **n = 6,405** | **n = 6,405** | **P**^a^ | **n = 19,467** | **n = 19,467** | **P**^a^ |
| Age category, *n(%)*  18 to 49 yr  50 to 64 yr  ≥65 yr | 2,176 (34.0)  1,869 (29.2)  2,360 (36.8) | 2,170 (33.9)  1,869 (29.2)  2,366 (36.9) | NA | 6,261 (32.2)  7,349 (37.8)  5,857 (30.1) | 6,266 (32.2)  7,345 (37.7)  5,856 (30.1) | NA |
| Male, *n(%)* | 3,133 (48.9) | 2,762 (43.1) | <0.001 | 15,451 (79.4) | 7,382 (37.9) | <0.001 |
| Median neighborhood income quintile, *n(%)*  Missing  1 - lowest  2  3  4  5 | ≤5 (S)  1,210-1,214 (S)  1,253 (19.6)  1,291 (20.2)  1,339 (20.9)  1,306 (20.4) | ≤5 (S)  1,210-1,214 (S)  1,253 (19.6)  1,291 (20.2)  1,339 (20.9)  1,306 (20.4) | NA | 21 (0.1)  2,770 (14.2)  3,393 (17.4)  3,795 (19.5)  4,398 (22.6)  5,090 (26.1) | 21 (0.1)  2,770 (14.2)  3,393 (17.4)  3,795 (19.5)  4,398 (22.6)  5,090 (26.1) | NA |
| Rural home location, *n(%)* | 1,011 (15.8) | 1,011 (15.8) | NA | 1,936 (9.9) | 1,936 (9.9) | NA |
| Resource utilization band^b^, *n(%)*  0 - lowest  1  2  3  4  5 | 0 (0.0)  ≤5 (S)  242-246 (S)  2,586 (40.4)  1,881 (29.4)  1,693 (26.4) | 0 (0.0)  ≤5 (S)  242-246 (S)  2,586 (40.4)  1,881 (29.4)  1,693 (26.4) | NA | 0 (0.0)  0 (0.0)  1,603 (8.2)  10,516 (54.0)  3,758 (19.3)  3,590 (18.4) | 0 (0.0)  0 (0.0)  1,603 (8.2)  10,516 (54.0)  3,758 (19.3)  3,590 (18.4) | NA |
| Charlson Comorbidity Index, *n*(%)  0  1  ≥2 | 4,912 (76.7)  455 (7.1)  1,038 (16.2) | 4,907 (76.6)  498 (7.8)  1,000 (15.6) |  | 15,883 (81.6)  864 (4.4)  2,720 (14.0) | 16,004 (82.2)  987 (5.1)  2,476 (12.7) |  |
| Mortality Risk Score^c^, *mean ± SD* |  |  |  |  |  |  |
| Year of admission, *n(%)*  2005  2006  2007  2008  2009  2010  2011  2012  2013  2014  2015 | 581 (9.1)  598 (9.3)  632 (9.9)  623 (9.7)  606 (9.5)  524 (8.2)  523 (8.2)  527 (8.2)  593 (9.3)  617 (9.6)  581 (9.1) | 581 (9.1)  598 (9.3)  632 (9.9)  623 (9.7)  606 (9.5)  524 (8.2)  523 (8.2)  527 (8.2)  593 (9.3)  617 (9.6)  581 (9.1) | NA | 2,070 (10.6)  1,981 (10.2)  1,794 (9.2)  1,771 (9.1)  1,829 (9.4)  1,746 (9.0)  1,683 (8.6)  1,749 (9.0)  1,559 (8.0)  1,652 (8.5)  1,633 (8.4) | 2,070 (10.6)  1,981 (10.2)  1,794 (9.2)  1,771 (9.1)  1,829 (9.4)  1,746 (9.0)  1,683 (8.6)  1,749 (9.0)  1,559 (8.0)  1,652 (8.5)  1,633 (8.4) | NA |
| Admission to a teaching hospital, *n(%)* | 1,351 (21.1) | 2,355 (36.8) | <0.001 | 3,003 (15.4) | 6,275 (32.2) | <0.001 |
| Surgical procedures with ≥8 OHIP anesthesia basic units, *n(%)* | 1,081 (16.9) | 1,081 (16.9) | NA | 1,593 (8.2) | 1,593 (8.2) | NA |
| Admitted to a special care unit prior to surgery, *n(%)* |  |  |  |  |  |  |
| Days from admission to surgery, *mean ± SD* | 0.14 ± 0.36 | 0.04 ± 0.21 | <0.001 | 1.18 ± 0.64 | 0.09 ± 0.30 | <0.001 |
| Length of hospital stay, *mean ± SD* | 3.64 ± 5.57 | 3.39 ± 4.84 | 0.006 | 4.85 ± 4.96 | 3.05 ± 2.91 | <0.001 |

SD, standard deviation; OHIP, Ontario Health Insurance Plan; S, suppressed percentage (cell counts <6 cannot be reported)

^a^P values not reported for variables used in exacting matching of study groups

^b^Resource utilization band is a ranking system of overall morbidity based on the Johns Hopkins Adjusted Clinical Group case-mix system

^c^Mortality Risk Score based on the Johns Hopkins Adjusted Clinical Group case-mix system
