# Supplementary material for: Hospital admission on weekends for patients who have surgery and 30-day mortality in Ontario, Canada: A matched cohort study
Source: PLoS Med. 2019 Jan 29;16(1):e1002731. doi: 10.1371/journal.pmed.1002731 (PMC6350956; doi:10.1371/journal.pmed.1002731)
Supplement: S8 Table — (DOCX) [file pmed.1002731.s010.docx]

**S8 Table.** Characteristics of urgent admissions included in the matched cohorts, classified by day of surgery (weekend or weekday).

| **Characteristic** | **Weekend admission and surgery** | **Weekday admission and surgery** |  | **Weekend admission and weekday surgery** | **Weekday admission and surgery** |  |
| --- | --- | --- | --- | --- | --- | --- |
|  | **n = 79,339** | **n = 79,339** | **P**^a^ | **n = 53,890** | **n = 53,890** | **P**^a^ |
| Age category, *n(%)*  18 to 49 yr  50 to 64 yr  ≥65 yr | 35,147 (44.3)  17,491 (22.0)  26,701 (33.7) | 35,121 (44.3)  17,503 (22.1)  26,715 (33.7) | NA | 14,285 (26.5)  11,533 (21.4)  28,072 (52.1) | 14,265 (26.5)  11,556 (21.4)  28,069 (52.1) | NA |
| Male, *n(%)* | 38,265 (48.2) | 38,136 (48.1) | <0.001 | 24,424 (45.3) | 24,707 (45.8) | <0.001 |
| Median neighborhood income quintile, *n(%)*  Missing  1 - lowest  2  3  4  5 | 39 (0.0)  16,749 (21.1)  16,239 (20.5)  15,678 (19.8)  15,867 (20.0)  14,767 (18.6) | 39 (0.0)  16,749 (21.1)  16,239 (20.5)  15,678 (19.8)  15,867 (20.0)  14,767 (18.6) | NA | 35 (0.1)  12,285 (22.8)  10,996 (20.4)  10,393 (19.3)  10,542 (19.6)  9,639 (17.9) | 35 (0.1)  12,285 (22.8)  10,996 (20.4)  10,393 (19.3)  10,542 (19.6)  9,639 (17.9) | NA |
| Rural home location, *n(%)* | 5,253 (6.6) | 5,253 (6.6) | NA | 3,722 (6.9) | 3,722 (6.9) | NA |
| Resource utilization band^b^, *n(%)*  0 - lowest  1  2  3  4  5 | ≤5 (S)  7-11 (S)  5,878 (7.4)  29,584 (37.3)  21,318 (26.9)  22,548 (28.4) | ≤5 (S)  7-11 (S)  5,878 (7.4)  29,584 (37.3)  21,318 (26.9)  22,548 (28.4) | NA | 0 (0.0)  0 (0.0)  1,525 (2.8)  13,734 (25.5)  14,607 (27.1)  24,024 (44.6) | 0 (0.0)  0 (0.0)  1,525 (2.8)  13,734 (25.5)  14,607 (27.1)  24,024 (44.6) | NA |
| Charlson Comorbidity Index, *n*(%)  0  1  ≥2 | 64,240 (81.0)  5,551 (7.0)  9,548 (12.0) | 63,053 (79.5)  5,573 (7.0)  10,713 (13.5) | <0.001 | 36,089 (67.0)  5,496 (10.2)  12,305 (22.8) | 37,790 (70.1)  5,200 (9.6)  10,900 (20.2) | <0.001 |
| Mortality Risk Score^c^, *mean ± SD* |  |  |  |  |  |  |
| Year of admission, *n(%)*  2005  2006  2007  2008  2009  2010  2011  2012  2013  2014  2015 | 7,141 (9.0)  6,887 (8.7)  6,983 (8.8)  7,036 (8.9)  6,956 (8.8)  7,072 (8.9)  7,239 (9.1)  7,351 (9.3)  7,561 (9.5)  7,611 (9.6)  7,502 (9.5) | 7,141 (9.0)  6,887 (8.7)  6,983 (8.8)  7,036 (8.9)  6,956 (8.8)  7,072 (8.9)  7,239 (9.1)  7,351 (9.3)  7,561 (9.5)  7,611 (9.6)  7,502 (9.5) | NA | 4,403 (8.2)  4,439 (8.2)  4,566 (8.5)  4,678 (8.7)  4,822 (8.9)  4,675 (8.7)  4,971 (9.2)  5,137 (9.5)  5,255 (9.8)  5,542 (10.3)  5,402 (10.0) | 4,403 (8.2)  4,439 (8.2)  4,566 (8.5)  4,678 (8.7)  4,822 (8.9)  4,675 (8.7)  4,971 (9.2)  5,137 (9.5)  5,255 (9.8)  5,542 (10.3)  5,402 (10.0) | NA |
| Admission to a teaching hospital, *n(%)* | 23,154 (29.2) | 24,280 (30.6) | <0.001 | 20,272 (37.6) | 16,948 (31.4) | <0.001 |
| Surgical procedures with ≥8 OHIP anesthesia basic units, *n(%)* | 5,694 (7.2) | 5,694 (7.2) | NA | 5,639 (10.5) | 5,639 (10.5) | NA |
| Admitted to a special care unit prior to surgery, *n(%)* |  |  |  |  |  |  |
| Days from admission to surgery, *mean ± SD* | 0.3 ± 0.4 | 0.4 ± 0.6 | <0.001 | 0.5 ± 0.6 | 2.2 ± 1.3 | <0.001 |
| Length of hospital stay, *mean ± SD* | 5.4 ± 10.3 | 5.5 ± 11.6 | 0.006 | 9.0 ± 12.8 | 7.1 ± 13.8 | <0.001 |

SD, standard deviation; OHIP, Ontario Health Insurance Plan; S, suppressed percentage (cell counts <6 cannot be reported)

^a^P values not reported for variables used in exacting matching of study groups

^b^Resource utilization band is a ranking system of overall morbidity based on the Johns Hopkins Adjusted Clinical Group case-mix system

^c^Mortality Risk Score based on the Johns Hopkins Adjusted Clinical Group case-mix system
