# Supplementary material for: Hospital admission on weekends for patients who have surgery and 30-day mortality in Ontario, Canada: A matched cohort study
Source: PLoS Med. 2019 Jan 29;16(1):e1002731. doi: 10.1371/journal.pmed.1002731 (PMC6350956; doi:10.1371/journal.pmed.1002731)
Supplement: S9 Table — (DOCX) [file pmed.1002731.s011.docx]

**S9 Table.** Adjusted odds ratios of 30-day all-cause mortality for patients admitted on weekends who had noncardiac surgery compared with reference admissions, measured from day of admission and from day of surgery.

|  | **30-day all-cause mortality** | |
| --- | --- | --- |
|  | **From date of admission** | **From date of surgery** |
| **Admission type** |  |  |
| All weekend admissions | 1.05 (1.00 to 1.11) | 1.08 (1.03 to 1.13) |
| Weekend admission and weekend surgery  Urgent  Elective  Subtotal (urgent + elective) | 1.02 (0.95 to 1.09)  3.30 (1.98 to 5.49)^a^  1.04 (0.97 to 1.11) | \| 1.01 (0.94 to 1.08) \| \| --- \| \| 3.30 (1.98 to 5.49)^a^ \| \| 1.03 (0.96 to 1.11) \| |
| Weekend admission and weekday surgery  Urgent  Elective  Subtotal (urgent + elective) | 1.05 (0.98 to 1.12)  2.70 (1.81 to 4.03)^b^  1.06 (1.00 to 1.14) | \| 1.10 (1.03 to 1.17) \| \| --- \| \| 2.80 (1.87 to 4.17)^b^ \| \| 1.11 (1.04 to 1.18) \| |

Variables used for exact matching were age in years, anesthesia basic unit value for

the surgical procedure, median neighborhood household income, resource utilization band, rural home location, year of admission, and urgency of admission. Covariates adjusted for in models were Charlson Comorbidity Index, Local Health Integrated Network, sex, teaching hospital status, mortality risk score, preoperative special care unit admission, and responsible surgical service.

^a^Not adjusted for Local Health Integrated Network and responsible surgical service due to lack of model convergence.

^b^Not adjusted for responsible surgical service due to lack of model convergence.
